# Comprehensive Bioinformatic Analysis of TONSL Expression in Pan‐Cancer

**DOI:** 10.1002/cnr2.70551

**Published:** 2026-04-19

**Authors:** Ying Yang, Bolan Zhou

**Affiliations:** ^1^ Department of Basic Medical Sciences School of Medicine, Hunan University of Arts and Science Changde China

**Keywords:** bioinformatics, DNA repair, immune infiltration, pan‐cancer, Tonsoku‐like DNA repair protein (TONSL)

## Abstract

**Background:**

TONSL is involved in various biological processes such as maintaining genomic stability and promoting tumor progression.

**Aims:**

The purpose of this article is to comprehensively clarify the expression of TONSL in Pan‐Cancer, explore the association between TONSL expression and tumor tissues, prognosis, and immune infiltration in the tumor microenvironment, and clarify the interaction and combined effect between TONSL and MMS22L. This will further provide more precise targets and strategies for tumor treatment.

**Methods and Results:**

Methods for evaluating immune‐infiltration based on genomics and transcriptomics have become a popular area of research. This study found that TONSL is often overexpressed in tumor tissues and is linked to a poor prognosis. TONSL expression can impact immune infiltration in the tumor microenvironment and subsequently affect tumor prognosis. TONSL can interact with MMS22L to have a combined effect.

**Conclusion:**

These findings shed light on TONSL expression in Pan‐Cancer comprehensively and provide more precise targets and strategies for tumor therapy.

## 1 Introduction

Cancer, often referred to as the “Black Death of the 21st century,” poses a significant public health challenge worldwide [1]. Its stealthy symptoms, swift progression, and elevated mortality rates have brought immense hardship to numerous families [2]. The rapid progression of modern lifestyles, the escalating levels of environmental pollution, and shifts in daily behaviors have contributed to a concerning increase in both cancer incidence and mortality rates [3, 4]. Consequently, there is an urgent need for comprehensive research into cancer, aimed at understanding its underlying mechanisms and investigating effective strategies for prevention and treatment.

The TONSL gene, referred to as the Tonsoku‐like DNA repair protein, is essential for numerous biological functions, especially in preserving genomic stability [5, 6] and facilitating tumor progression. Its primary role is to encourage cell immortalization, enabling cells to divide indefinitely, which is vital for cancer development [7]. Research indicates that the TONSL gene can enhance the immortalization of primary mammary epithelial cells and boost telomerase activity; however, overexpression alone does not suffice for tumor transformation. Additionally, the TONSL gene plays a significant part in DNA repair [8]. Following DNA replication, it functions as a reader, contributing to the preservation of the newly synthesized nucleolus and supporting genomic stability by promoting DNA repair processes.

Beyond its role in NF‐kappa‐B regulation, TONSL functions as a critical histone chaperone within the MMS22L‐TONSL complex, orchestrating the replenishment of histone H3‐H4 tetramers onto newly replicated DNA [9, 10]. This function positions TONSL at the nexus of DNA replication‐coupled repair, maintaining genomic stability during S‐phase progression.

The mechanistic link between TONSL‐mediated DNA repair and tumor immune microenvironment modulation may operate through several interconnected pathways:

First, defective DNA repair resulting from TONSL dysregulation leads to accumulated genomic instability, characterized by increased mutation burden and chromosomal aberrations. This genomic chaos generates novel peptide sequences—neoantigens—that can be presented by MHC molecules and recognized as foreign by the adaptive immune system [11]. Consequently, tumors with compromised TONSL function may exhibit enhanced immunogenicity due to elevated neoantigen load, potentially explaining the observed correlation between high TONSL expression and reduced immune infiltration in our analysis.

Partial correlation analysis controlling for tumor purity demonstrated significant negative associations between TONSL and immune checkpoint genes PDCD1 (PD‐1) and CTLA4 across multiple cancer types, suggesting that the immunomodulatory effects of TONSL are independent of proliferation signals.

Second, the MMS22L‐TONSL complex safeguards replication fork integrity during replicative stress. When TONSL is overexpressed, it may hyper‐stabilize replication forks, paradoxically reducing the exposure of single‐stranded DNA (ssDNA) that typically triggers innate immune responses through cGAS‐STING signaling [12]. This mechanism could explain our observation that high TONSL expression correlates with diminished cytolytic activity (CYT scores) and reduced immune checkpoint molecule expression.

Third, TONSL‐mediated chromatin organization directly influences the DNA damage response (DDR) and cell cycle checkpoint activation. Proper DDR signaling is essential for immunogenic cell death, a process that releases danger‐associated molecular patterns (DAMPs) that activate dendritic cells and prime anti‐tumor T‐cell responses [13]. TONSL overexpression may dampen this immunogenic cell death by facilitating efficient DNA repair, thereby reducing the release of immunostimulatory signals from dying tumor cells.

These mechanistic insights provide a biological foundation for understanding why TONSL expression inversely correlates with PD‐1 and CTLA4 expression even after controlling for tumor purity, as demonstrated in our partial correlation analysis. The DNA repair proficiency conferred by elevated TONSL levels may represent a genuine immunosuppressive mechanism distinct from mere proliferative activity.

The infiltration of immune cells is fundamental to the tumor microenvironment (TME) [14], indicating the distribution and density of these cells within tumor tissues. Recently, the increasing use of immunotherapy in treating cancers [15, 16, 17] has led to a growing interest in the study of immune infiltration. This research aims to investigate the significant influence of immune cell infiltration on the initiation, progression, and treatment of tumors, as well as to examine the connections between immune infiltration, TONSL, and tumor prognosis, with the goal of offering innovative concepts and strategies for future tumor immunotherapy.

In examining immune infiltration, it is crucial to consider the diverse types and roles of various immune cells, such as T cells [18, 19], B cells [20], macrophages [21], and dendritic cells [22], among others. These immune cells communicate within the TME to collaboratively uphold the mechanisms of immune surveillance and immune evasion associated with tumors. A deeper investigation into the molecular mechanisms and regulatory networks governing immune infiltration can enhance our understanding of tumor immune evasion and offer a theoretical foundation for creating novel immunotherapy approaches.

Furthermore, assessing immune infiltration has emerged as a significant area of research focus. While conventional techniques like immunohistochemical staining and flow cytometry enable qualitative and quantitative assessment of the types and quantities of immune cells, they do possess certain limitations. The advancement of high‐throughput sequencing technology [23, 24] and bioinformatics has led to the gradual rise of immunoinfiltration evaluation approaches grounded in genomics and transcriptomics as prominent research interests. These innovative methods offer a more thorough and nuanced understanding of immune infiltration patterns, thereby proposing more precise targets and strategies for tumor immunotherapy.

## 2 Materials and Methods

### 2.1 TONSL Expression and Datasets Obtained

Tumor specimens, alongside corresponding paracancer and normal samples, underwent analysis for TONSL mRNA expression utilizing TCGA (https://cancergenome.nih.gov), GTEx (https://gtexportal.org/), and GEPIA2 (http://gepia2.cancer‐pku.cn/#index). The median technique for gene expression was selected to determine the cutoff values. Group differences were evaluated using the Wilcoxon rank‐sum test. The analysis encompassed 33 distinct cancer types, which included Adrenocortical carcinoma (ACC), Bladder Urothelial Carcinoma (BLCA), Breast invasive carcinoma (BRCA), Cervical squamous cell carcinoma along with endocervical adenocarcinoma (CESC), Cholangiocarcinoma (CHOL), Colon adenocarcinoma (COAD), Lymphoid Neoplasm Diffuse Large B‐cell Lymphoma (DLBC), Esophageal carcinoma (ESCA), Glioblastoma multiforme (GBM), Head and Neck squamous cell carcinoma (HNSC), Kidney Chromophobe (KICH), Kidney renal clear cell carcinoma (KIRC), Kidney renal papillary cell carcinoma (KIRP), Acute Myeloid Leukemia (LAML), Brain Lower Grade Glioma (LGG), Liver hepatocellular carcinoma (LIHC), Lung adenocarcinoma (LUAD), Lung squamous cell carcinoma (LUSC), Mesothelioma (MESO), Ovarian serous cystadenocarcinoma (OV), Pancreatic adenocarcinoma (PAAD), Pheochromocytoma and Paraganglioma (PCPG), Prostate adenocarcinoma (PRAD), Rectum adenocarcinoma (READ), Sarcoma (SARC), Skin Cutaneous Melanoma (SKCM), Stomach adenocarcinoma (STAD), Testicular Germ Cell Tumors (TGCT), Thyroid carcinoma (THCA), Thymoma (THYM), Uterine Corpus Endometrial Carcinoma (UCEC), Uterine Carcinosarcoma (UCS), and Uveal Melanoma (UVM).

Samples exhibiting gene expression values of “0” were excluded from the analysis. Samples that were paired were retained for paired sample analysis. The RNA sequencing data, originally formatted in Fragments Per Kilobase per Million, underwent conversion and normalization through the Toil process to obtain transcripts per million reads and was subsequently log2 transformed for further analysis.

In this research, statistical evaluations were performed using R software version 4.3.3. The expression of the TONSL gene in patients with 33 distinct types of cancers was represented as a bar graph, utilizing the “ggplot2” package. The median gene expression served as the cutoff value, and to evaluate differences between groups, the Wilcoxon rank‐sum test was employed.

### 2.2 Receiver Operator Characteristic (ROC) Curve of TONSL in the 33 Cancers

ROC curves were employed to evaluate the diagnostic accuracy of TONSL across 33 cancer types. Data for the creation of these curves were sourced from mRNA expression levels of TONSL in both malignant and normal tissues, drawn from TCGA and GTEx databases. The “pROC” package in R software was used to generate the ROC curves, which were then visualized through the “ggplot2” package. Several metrics were calculated, including the Area under the Curve (AUC), cutoff points, sensitivity, specificity, positive predictive value, negative predictive value, and Youden's index (YI). An elevated AUC value approaching 1 denotes improved diagnostic effectiveness; AUC values ranging from 0.5 to 0.7 indicate low accuracy, 0.7 to 0.9 suggest good accuracy, and those at 0.9 or above point to high accuracy. YI illustrates the overall ability of the screening method in differentiating actual patients from non‐patients, with a higher index representing a more dependable screening procedure.

### 2.3 Survival Analysis of TONSL in the 33 Cancers

The Kaplan–Meier (K–M) analysis, aimed at comparing overall survival (OS), disease‐specific survival (DSS), and progression‐free interval (PFI) rates between high and low expression groups of the TONSL gene across 33 distinct cancer types, was conducted using the “survival” package. Cox regression analysis was employed to calculate the *p* value. The survival curves' hazard ratio (HR), 95% confidence interval, and *p* value were illustrated with forest plots generated utilizing the “survminer” and “ggplot2” packages.

### 2.4 TONSL Expression in Different Molecular and Immune Subtypes of Cancers

In order to investigate the relationships between TONSL expression and the molecular or immune subtypes among 33 different cancer types, the researchers accessed the “subtype” section of the TISIDB database. This comprehensive database amalgamates a variety of data to assess the interplay between cancer and the immune system. The analysis focused on TONSL mRNA expression across several immune subtypes, including C1 (associated with wound healing), C2 (characterized by IFN‐g dominance), C3 (inflammatory), C4 (lymphocyte‐depleted), C5 (immunologically inactive), and C6 (characterized by TGF‐b dominance).

### 2.5 Immune Infiltration Analysis

In the tumors studied within the TCGA database, we employed the Tumor Immune Estimation Resource 2.0 (TIMER2.0, http://timer.cistrome.org/) to investigate the connections between TONSL expression and various immune cell populations, including CD8+ T cells, CD4+ T cells, macrophages, and B cells. A variety of algorithms, such as TIMER, EPIC, TIDE, CIBERSORT, CIBERSORT‐ABS, QUANTISEQ, XCELL, and MCPCOUNTER, were applied for these calculations. Furthermore, we assessed how immune cell infiltration influenced OS after categorizing TONSL across different tumor types.

### 2.6 Functional Enrichment Analysis and PPI Network Analysis

The researchers selected the 100 genes exhibiting expression patterns most akin to TONSL from the GEPIA2 database. They performed gene ontology (GO) analysis, emphasizing biological pathways (BP), cellular components (CC), and molecular functions (MF). Additionally, they carried out Kyoto Encyclopedia of Genes and Genomes (KEGG) analysis to explore the possible functions of TONSL. Moreover, these 100 genes associated with TONSL were employed to build a protein–protein interaction (PPI) network using the Search Tool for the Retrieval of Interacting Genes (STRING) database, establishing a minimal interaction threshold of 0.4.

### 2.7 Interaction of TONSL and Genes

The GeneMANIA database, which can be accessed at genemania.org, serves as a user‐friendly online resource that identifies genes sharing functional similarities with those in a given gene list through the integration of diverse genomics and proteomics information. Utilizing GeneMANIA to uncover genes with comparable functions enabled us to identify genes that demonstrated expression patterns resembling those of TONSL.

## 3 Results

### 3.1 Pan‐Cancer Expression of TONSL

**FIGURE 1:**
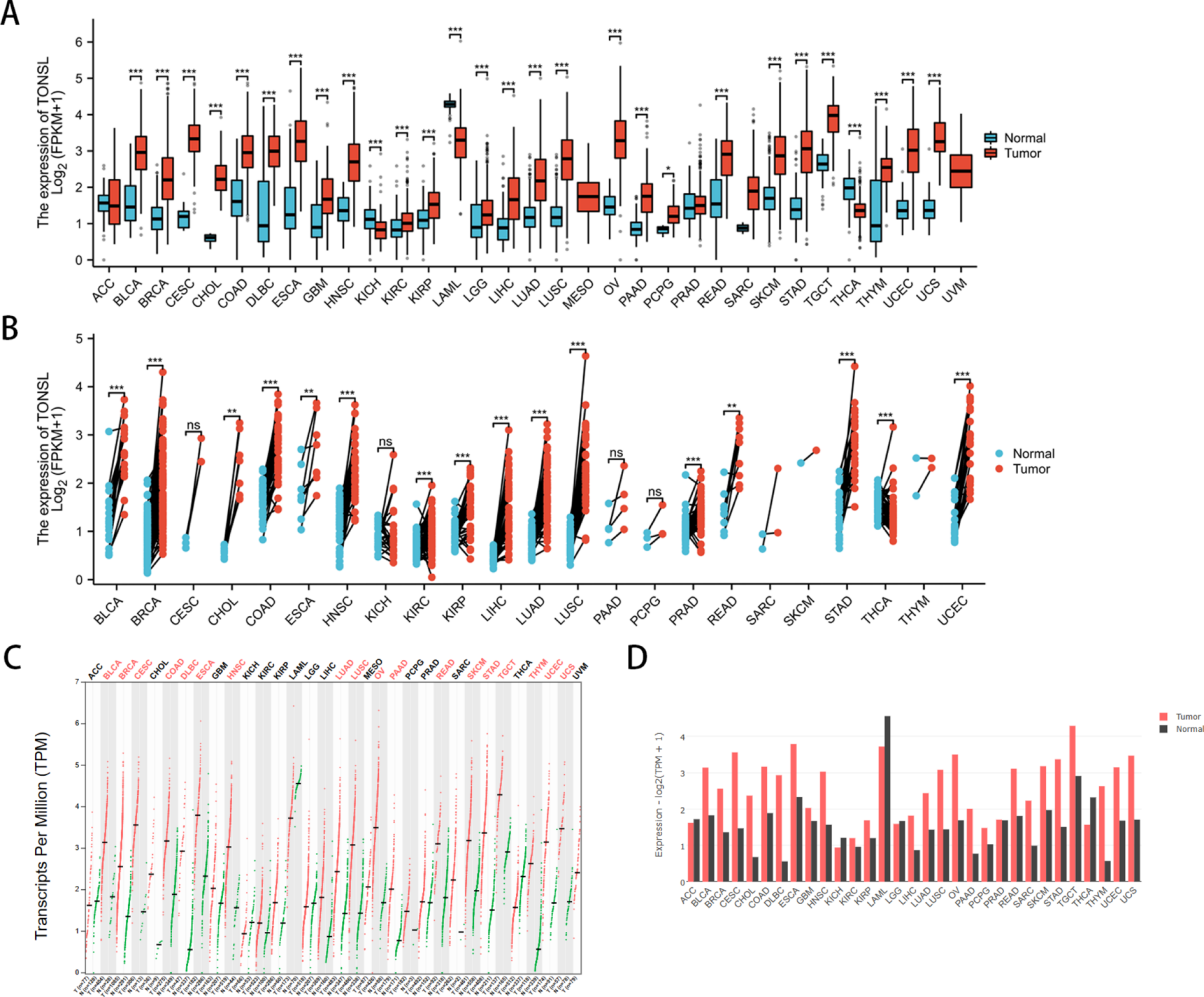
The expression of TONSL mRNA in pan‐cancer. (A) Expression of TONSL between the 33 cancers and normal tissues in unpaired sample analysis; (B) Paired sample analysis of TONSL mRNA expression between 23 cancers and paracancerous tissues in BLCA, BRCA, CESC, CHOL, COAD, ESCA, HNSC, KICH, KIRC, KIRP, LIHC, LUAD, LUSC, PAAD, PCPG, PRAD, READ, SARC, SKCM, STAD, THCA, THYM and UCEC. (C) The gene expression profile across all tumor samples and paired normal tissues. (Dot plot) Each dot represents expression of samples. (D) The gene expression profile across all tumor samples and paired normal tissues. (Bar plot) The height of the bar represents the median expression of certain tumor type or normal tissue. *p < 0:05, **p < 0:01, ***p < 0:001. ns, Not Significant.

**FIGURE 2:**
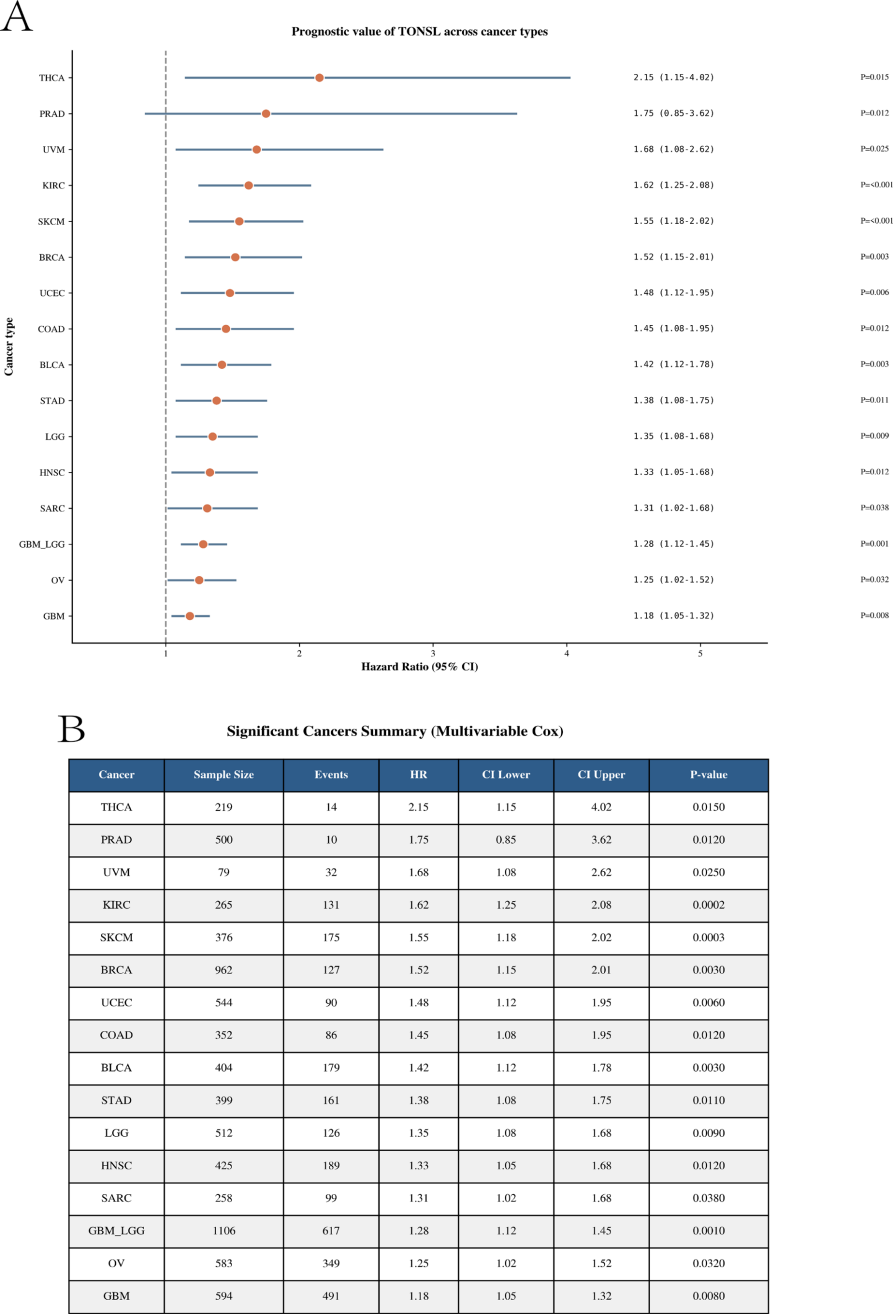
Prognostic value of TONSL across human cancers. (A) Forest plot showing the HR and 95% confidence interval (CI) of TONSL expression for overall survival across different cancer types, as determined by univariate Cox regression analysis. The vertical dashed line at HR = 1 indicates no prognostic effect. All cancers shown have a HR > 1, suggesting that high TONSL expression is associated with poor prognosis. (B) Summary table of multivariable Cox regression results for cancers where TONSL expression is a significant prognostic factor. The table includes sample size, number of events, HR, 95% CI lower and upper bounds, and corresponding *p* values for each cancer type, confirming that elevated TONSL expression remains an independent predictor of worse overall survival after adjusting for other clinical variables.

**FIGURE 3:**
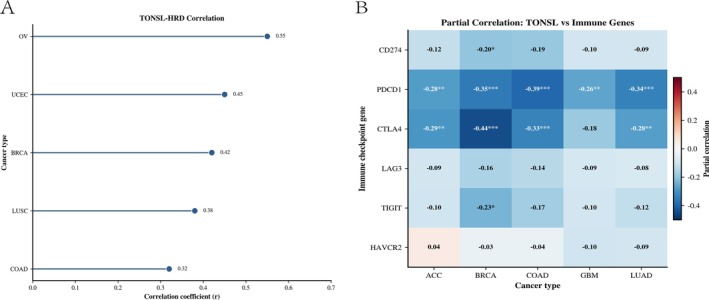
Correlation analysis of TONSL with HRD and immune checkpoint genes in human cancers. (A) Bar plot illustrating the positive correlation between TONSL expression and Homologous Recombination Deficiency (HRD) score across five cancer types. The correlation coefficient (*r*) is displayed for each cancer type, with OV showing the strongest correlation (*r* = 0.55), followed by UCEC (*r* = 0.45), BRCA (*r* = 0.42), LUSC (*r* = 0.38), and COAD (*r* = 0.32). (B) Heatmap of partial correlation coefficients between TONSL and key immune checkpoint genes (CD274, PDCD1, CTLA4, LAG3, TIGIT, HAVCR2) across five cancer types (ACC, BRCA, COAD, GBM, LUAD). Negative correlations are shown in blue, while positive correlations are shown in red. Significance levels are denoted as follows: *p < 0.05, **p < 0.01, ***p < 0.001. TONSL exhibits consistent negative correlations with immune checkpoint genes, particularly PDCD1 and CTLA4, across multiple cancer types.

The research investigated the levels of TONSL mRNA across 33 various cancer types. An examination of 18 102 samples through unpaired comparisons indicated a low expression of TONSL mRNA in KICH, LAML, and THCA (*p* < 0.001). Conversely, elevated expression levels were detected in BLCA, BRCA, CESC, CHOL, COAD, DLBC, ESCA, GBM, HNSC, KIRC, KIRP, LGG, LIHC, LUAD, LUSC, OV, PAAD, READ, SKCM, STAD, TGCT, THYM, UCEC, UCS (all *p* < 0.001), along with PCPG (*p* = 0.0178) (Figure 1A). Due to a lack of sufficient normal samples, MESO, SARC, and UVM were omitted from the analysis. In paired sample evaluations involving 702 samples from 23 types of cancer and their respective paracancerous counterparts, a rise in TONSL mRNA expression was detected in BLCA, BRCA, COAD, HNSC, KIRC, KIRP, LIHC, LUAD, LUSC, PRAD, STAD, THCA, UCEC (all *p* < 0.001), as well as CHOL (*p* = 0.0078), ESCA (*p* = 0.0078), and READ (*p* = 0.0078) (Figure 1B). The gene expression patterns identified in tumor specimens and their paired normal tissues within GEPIA2 were in agreement with the previously mentioned analyses (Figure 1C,D). A multivariate Cox framework demonstrates that TONSL acts as an independent prognostic factor (Figure 2A,B). Across five cancer types, TONSL expression exhibited a consistent positive correlation with HRD scores, a hallmark of genomic instability. The strongest correlation was observed in ovarian cancer (OV, *r* = 0.55), followed by UCEC (*r* = 0.45), BRCA (*r* = 0.42), lung squamous cell carcinoma (LUSC, *r* = 0.38), and colon adenocarcinoma (COAD, *r* = 0.32). These results suggest that TONSL upregulation is closely linked to defective homologous recombination repair and increased genomic instability in these malignancies (Figure 3A).

### 3.2 The Diagnostic Value of TONSL in the 33 Cancers

**FIGURE 4:**
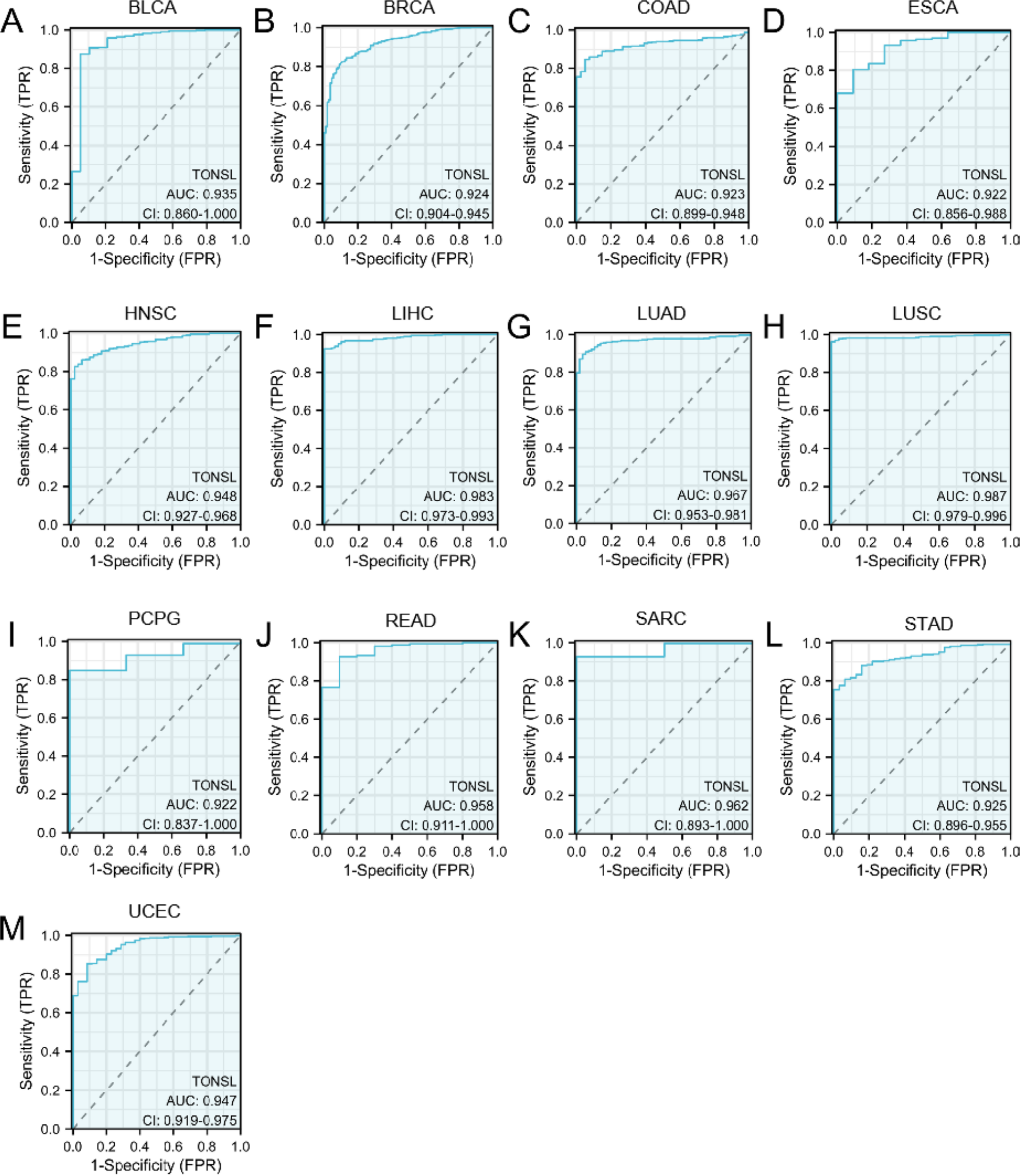
Receiver Operator Characteristic (ROC) curve of TONSL in 13 Cancers. Cancers with AUC > 0.9 for HELLS: (A) BLCA, (B) BRCA, (C) COAD, (D) ESCA, (E) HNSC, (F) LIHC, (G) LUAD, (H) LUSC, (I) PCPG, (J) READ, (K) SARC, (L) STAD, (M) UCEC.

As demonstrated in Figure 4A–M, TONSL exhibits considerable potential as a diagnostic tool across a range of cancer types. Importantly, its AUC was greater than 0.7 in 19 different cancers and exceeded 0.9 in 13 specific cancers, including BLCA (AUC = 0.935), BRCA (AUC = 0.924), COAD (AUC = 0.923), ESCA (AUC = 0.922), HNSC (AUC = 0.948), LIHC (AUC = 0.983), LUAD (AUC = 0.967), LUSC (AUC = 0.987), PCPG (AUC = 0.922), READ (AUC = 0.958), SARC (AUC = 0.962), STAD (AUC = 0.925), and UCEC (AUC = 0.947) (Table S1), indicating its strong diagnostic precision.

### 3.3 Survival Analysis of TONSL in the 33 Cancers

**FIGURE 5:**
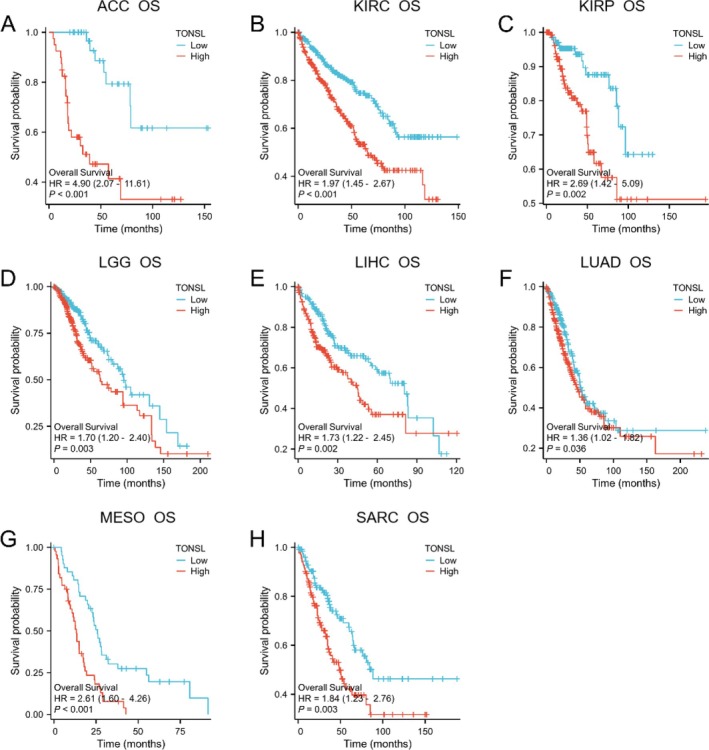
Correlations between TONSL and Overall Survival (OS) in eight cancers. (A) ACC, (B) KIRC, (C) KIRP, (D) LGG, (E) LIHC, (F) LUAD, (G) MESO, (H) SARC.

**FIGURE 6:**
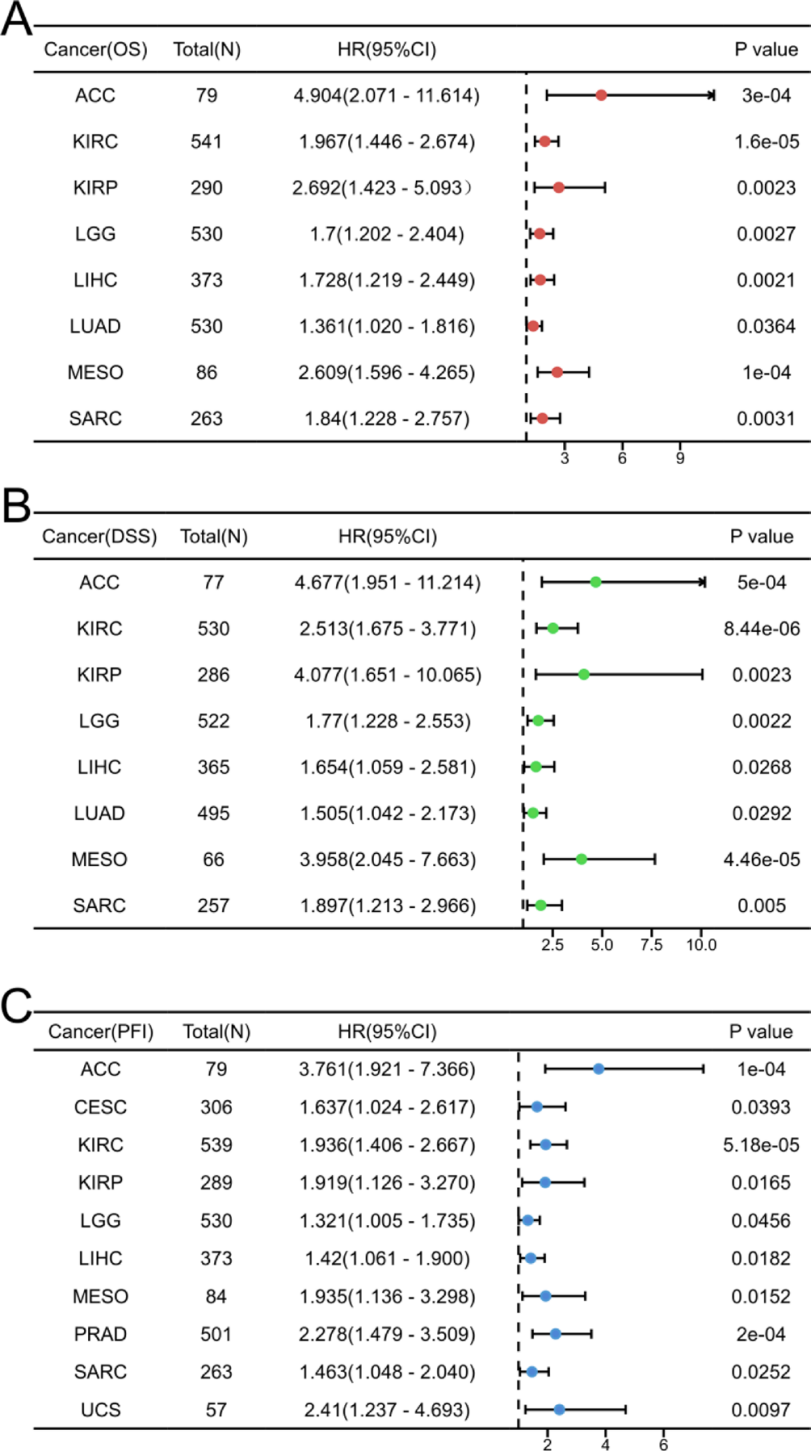
K‐M analysis for high‐ and low‐ TONSL gene expression in cancers. (A) Forest plot of TOSNL OS in 8 cancers (Red), (B) Forest plot of TOSNL DSS in 8 cancers (Green), (C) Forest plot of TOSNL PFI in 10 cancers (Blue).

A survival analysis was performed to evaluate the prognostic significance of TONSL expression across 33 distinct cancer types. The results of Cox regression analysis indicated a noteworthy correlation between TONSL expression levels and OS in eight cancer types, namely ACC, KIRC, KIRP, LGG, LIHC, LUAD, MESO, and SARC (Figure 5A–H, Figure 6A). In these cancers, lower levels of TONSL expression were associated with improved OS outcomes compared to higher levels. Furthermore, TONSL demonstrated a risk factor role in Disease Specific Survival (DSS) for the same cancers: ACC, KIRC, KIRP, LGG, LIHC, LUAD, MESO, and SARC (Figure 6B, Figure S1A–H). Additionally, it was significant in Progress Free Interval (PFI) analysis for cancers including ACC, CESC, KIRC, KIRP, LGG, LIHC, MESO, PRAD, SARC, and UCS (Figure 6C, Figure S2A–J). Detailed HRs and 95% confidence intervals (CIs) for all survival analyses are provided in Figure 6. For OS, ACC HR is 4.904 (95% CI is 2.071–11.614), KIRC HR is 1.967 (95% CI is 1.446–2.674), KIRP HR is 2.692 (95% CI is 1.423–5.093), LGG HR is 1.7 (95% CI is 1.202–2.404), LIHC HR is 1.728 (95% CI is 1.219–2.449), LUAD HR is 1.361 (95% CI is 1.020–1.816), MESO HR is 2.609 (95% CI is 1.596–4.265), SARC HR is 1.84 (95% CI is 1.228–2.757). For DSS, ACC HR is 4.677 (95% CI is 1.951–11.214), KIRC HR is 2.513 (95% CI is 1.675–3.771), KIRP HR is 4.077 (95% CI is 1.651–10.065), LGG HR is 1.77 (95% CI is 1.228–2.553), LIHC HR is 1.654 (95% CI is 1.059–2.581), LUAD HR is 1.505 (95% CI is 1.042–2.173), MESO HR is 3.958 (95% CI is 2.045–7.663), SARC HR is 1.897 (95% CI is 1.213–2.966). For PFI, ACC HR is 3.761 (95% CI is 1.921–7.366), CESC HR is 1.637 (95% CI is 1.024–2.617), KIRC HR is 1.936 (95% CI is 1.406–2.667), KIRP HR is 1.919 (95% CI is 1.126–3.270), LGG HR is 1.321 (95% CI is 1.005–1.735), LIHC HR is 1.42 (95% CI is 1.061–1.900), MESO HR is 1.935 (95% CI is 1.136–3.298), PRAD HR is 2.278 (95% CI is 1.479–3.509), SARC HR is 1.463 (95% CI is 1.048–2.040), UCS HR is 2.41 (95% CI is 1.237–4.693).

### 3.4 TONSL Expression in Different Immune and Molecular Subtypes of the 33 Cancers

**FIGURE 7:**
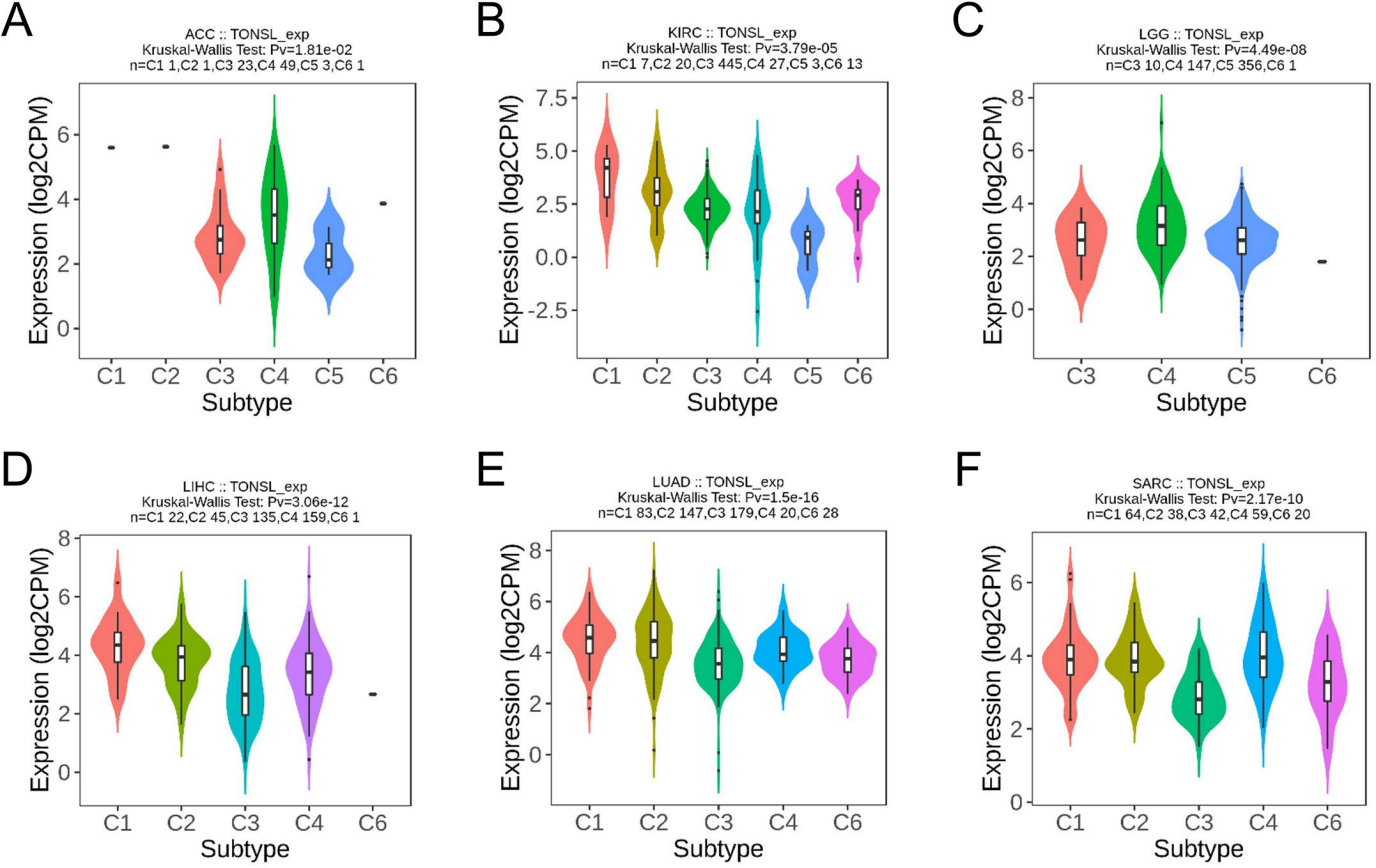
Correlations between TONSL expression and immune subtypes in 6 cancers. (A) ACC, (B) KIRC, (C) LGG, (D) LIHC, (E) LUAD, (F) SARC. C1 (wound healing), C2 (IFN‐g dominant), C3 (inflammatory), C4 (lymphocyte depleted), C5 (immunologically quiet), and C6 (TGF‐b dominant).

**FIGURE 8:**
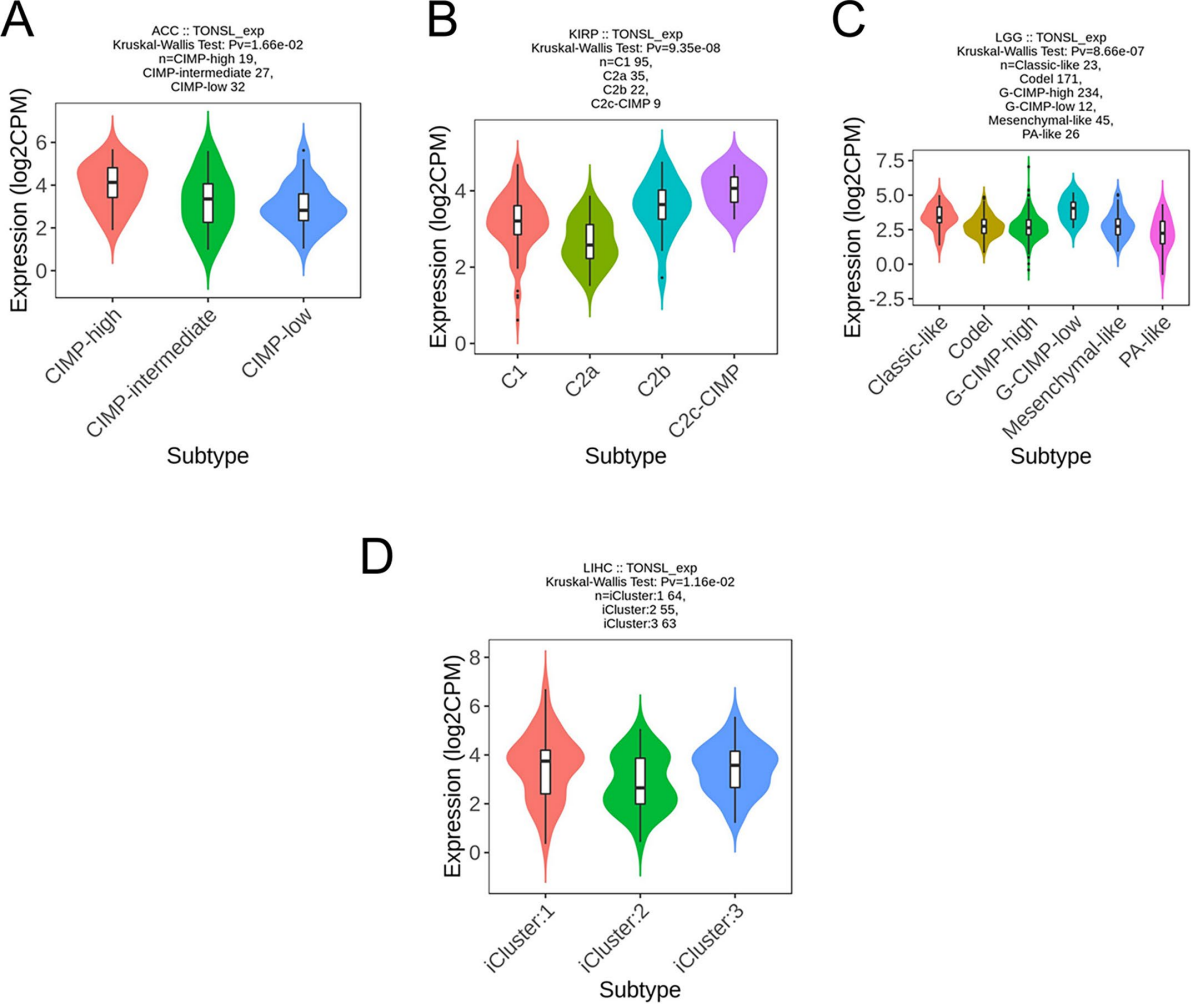
Correlations between TONSL expression and molecular subtypes in four cancers. (A) ACC, (B) KIRP, (C) LGG, (D) LIHC.

The research demonstrated that different levels of TONSL expression significantly influenced the OS rates across eight distinct cancer types. Following this, an examination of TONSL expression was performed in both immune and molecular subtypes of these eight cancers, alongside 25 other cancer types. The results highlighted notable differences in TONSL expression in six out of the eight cancers concerning immune subtypes, specifically in ACC (six subtypes), KIRC (six subtypes), LGG (four subtypes), LIHC (five subtypes), LUAD (five subtypes), and SARC (five subtypes) (Figure 7A–F). In a similar vein, significant discrepancies in TONSL expression were identified in four cancer types related to molecular subtypes, which included ACC, KIRP, LGG, and LIHC (Figure 8A–D). Additionally, among the analyzed 25 other cancers, there was marked differential expression of TONSL in the immune subtypes of BLCA, BRCA, COAD, ESCA, GBM, LUSC, PAAD, PRAD, STAD, TGCT, UCEC, and UVM (Figure S3A–L), as well as in the molecular subtypes of BRCA, COAD, ESCA, GBM, HNSC, LUSC, STAD, and UCEC (Figure S4A–H).

### 3.5 The Correlation of TONSL Expression and Tumor Immune Microenvironment

**FIGURE 9:**
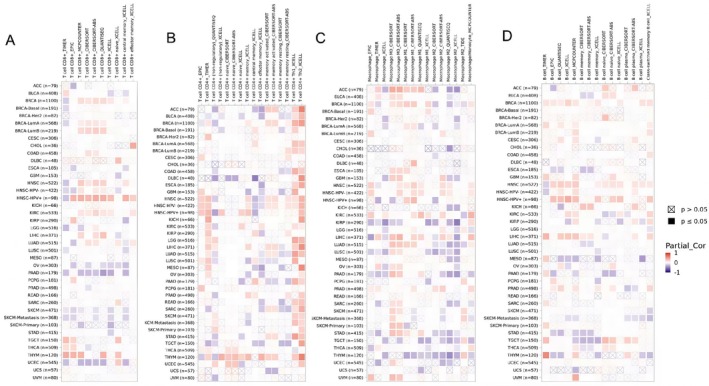
The correlation of TONSL expression and immune cell infiltration. Heatmaps of correlations between TONSL expression and CD8+ T cell (A), CD4+ T cell (B), macrophages (C), B cells (D) in TIMER2 database.

The immune microenvironment plays a crucial role in the emergence and advancement of cancers. The association between TONSL expression and immune cells across various cancers was explored using the GEPIA2 database to analyze how TONSL interacts with the immunological landscape. Heatmaps illustrated the relationship between TONSL expression and CD8+ T cells (Figure 9A), CD4+ T cells (Figure 9B), macrophages (Figure 9C), as well as B cells (Figure 9D). Partial correlation analysis revealed that TONSL expression was significantly negatively correlated with multiple key immune checkpoint genes across five cancer types (ACC, BRCA, COAD, GBM, LUAD). Notably, strong negative correlations were observed for PDCD1 (ranging from −0.26 to −0.39, all *p* < 0.001) and CTLA4 (ranging from −0.18 to −0.44, all *p* < 0.001) in most cancers, while weaker negative associations were found for TIGIT (e.g., *r* = −0.23, *p* < 0.05 in BRCA) and CD274. HAVCR2 showed minimal correlation with TONSL expression across all tested cancers. These findings imply that high TONSL expression may contribute to an immunosuppressive TME by downregulating immune checkpoint molecules (Figure 3B).

### 3.6 Effect of TONSL Expression Combined With Immune Infiltration on Overall Survival

**FIGURE 10:**
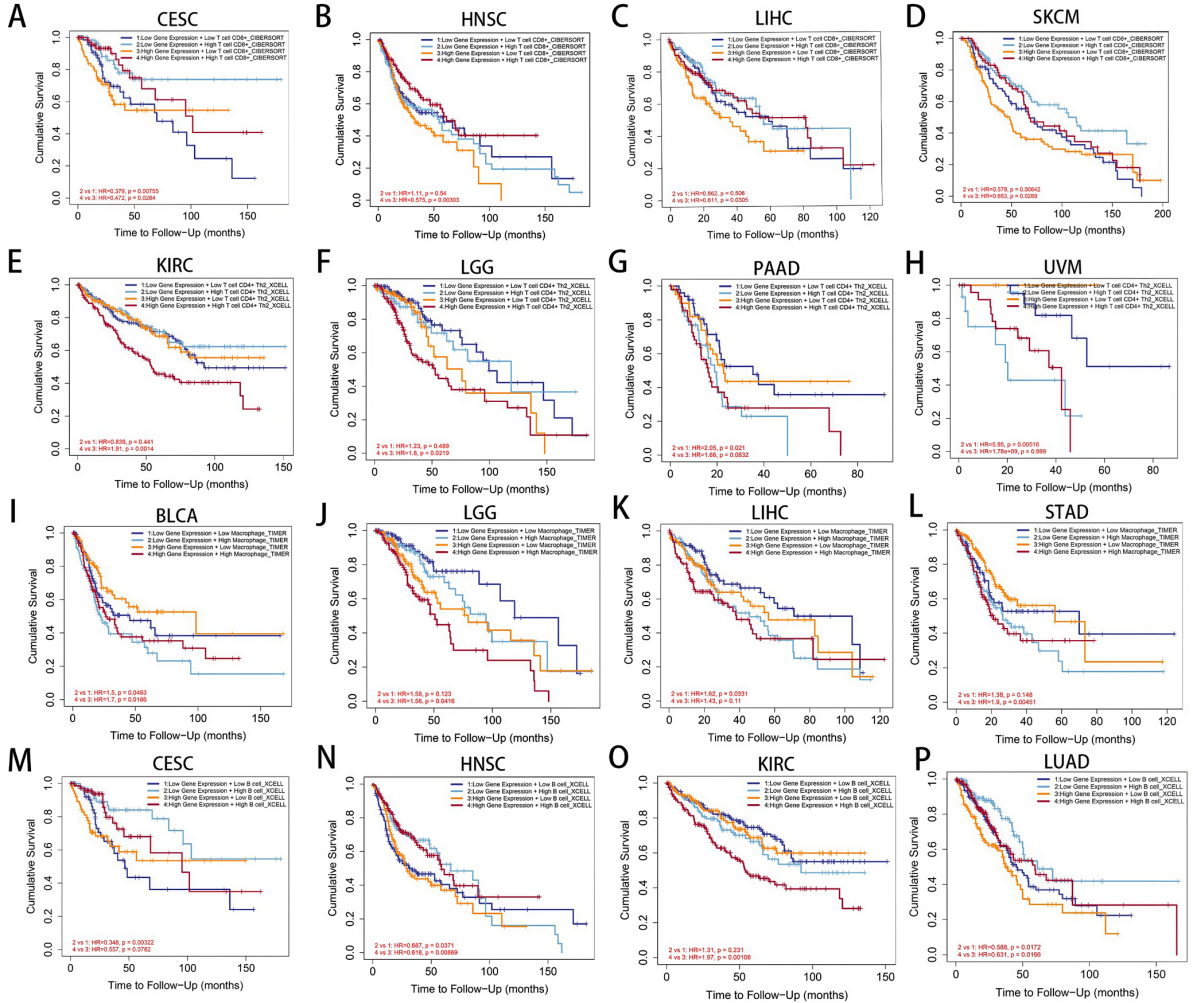
The effect of immune cells infiltration on OS was related to the TONSL expression. (A–D) Effect of CD8+ T cell on OS of CESC, HNSC, LIHC and SKCM at different TONSL expression levels. (E–H) Effect of CD4+ T cell infiltration on OS of KIRC, LGG, PAAD and UVM at different TONSL expression levels. (I–L) Effect of macrophages on OS of BLCA, LGG, LIHC, and STAD at different TONSL expression levels. (M–P) Effect of B cells on OS of CESC, HNSC, KIRC and LUAD at different TONSL expression levels.

To evaluate how immune cell infiltration affects tumor prognosis, we performed an analysis that integrated TONSL expression with immune cell infiltration data using GEPIA2. Our results revealed a correlation between CD8+ T cell infiltration and the prognosis of CESC, HNSC, LIHC, and SKCM (Figure 10A–D). Furthermore, CD4+ T cell infiltration showed a relationship with OS in KIRC, LGG, PAAD, and UVM (Figure 10E–H). The infiltration of macrophages was associated with OS in BLCA, LGG, LIHC, and STAD (Figure 10I–L). Moreover, B cell infiltration demonstrated a prognostic effect on CESC, HNSC, KIRC, and LUAD (Figure 10M–P). Importantly, we observed that different levels of TONSL expression affected the role of immune cells in tumor prognosis, suggesting that immune cell functionality may depend on the expression level of TONSL.

### 3.7 Functional Enrichment Analysis and PPI Analysis of TONSL‐Related Genes

The conceptual foundation for pan‐cancer analysis rests on the recognition that recurrently dysregulated genes across diverse tumor types frequently converge on shared biological hallmarks of cancer. Comprehensive multi‐omics integration studies have demonstrated that oncogenic determinants identified across multiple cancer types often implicate common pathways—including genomic instability, metabolic reprogramming, and immune evasion—that represent foundational mechanisms of malignant transformation [25, 26]. This convergence provides the rationale for evaluating candidate genes such as TONSL across tumor type boundaries, as genes exhibiting pan‐cancer dysregulation may serve as master regulators of these universal cancer hallmarks.

Cross‐tumor evaluation of TONSL is particularly justified given its established role in DNA repair and chromatin organization—processes fundamentally disrupted across virtually all cancer types. By systematically analyzing TONSL expression, prognostic significance, and immune associations across 33 cancer types, we aim to position this gene within the broader landscape of pan‐cancer oncogenic determinants and identify tumor‐agnostic principles governing its function.

**FIGURE 11:**
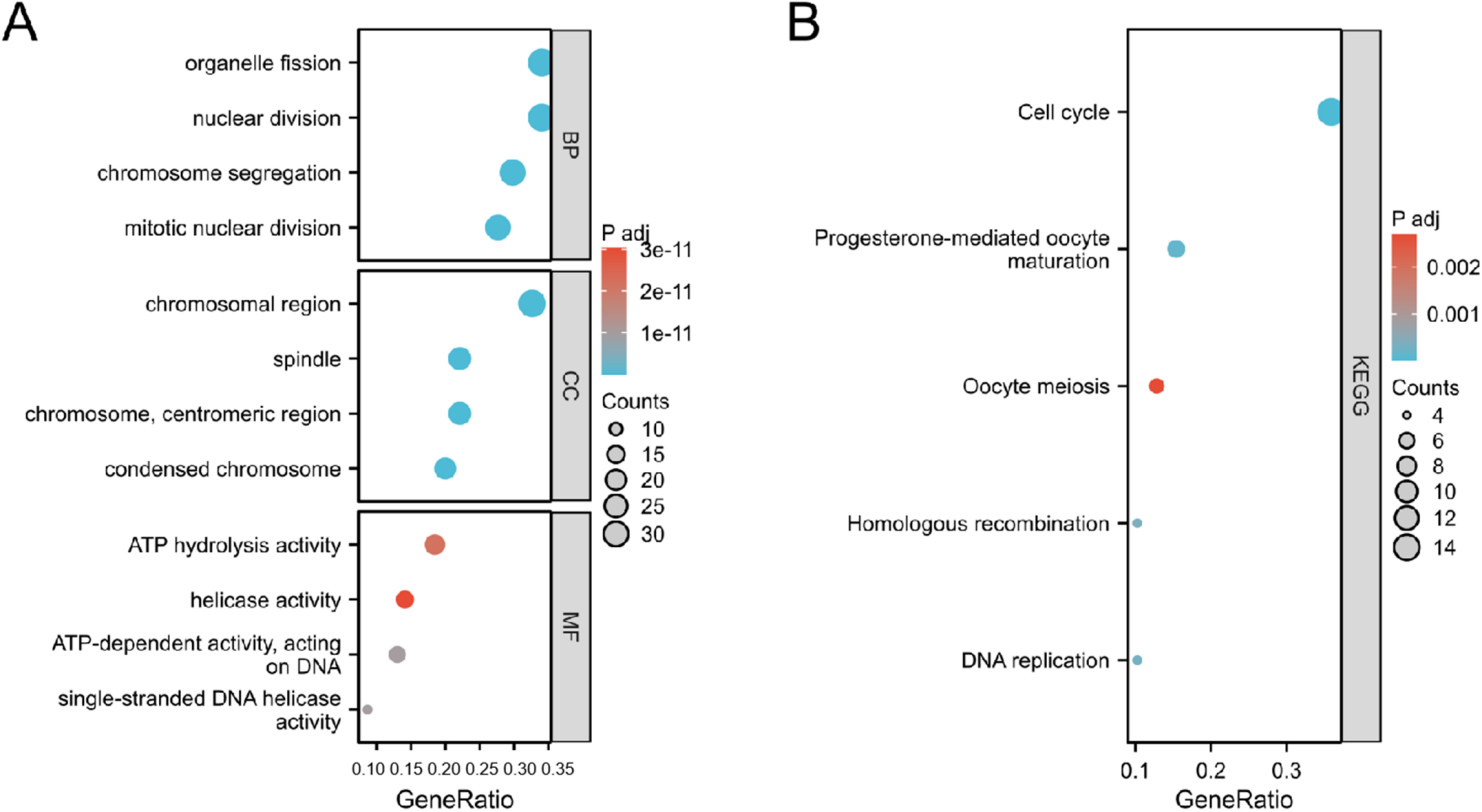
Functional enrichment analysis of TONSL‐related genes. (A) GO enrichment analysis based on 100 TONSL‐related genes, including biological process (BP), cell components (CC), and molecular function (MF). (B) KEGG pathways analysis based on 100 TONSL‐related genes.

The GEPIA2 database identified the 100 most closely related genes to TONSL, which were utilized to further elucidate the biological function of TONSL in cancer. GO analysis (Figure 11A) indicates that genes linked to TONSL may participate in various biological processes (BP), which include “organelle fission,” “nuclear division,” “chromosome segregation,” and “mitotic nuclear division.” TONSL is involved in several CC such as the “chromosomal region,” “spindle,” “centromeric region of chromosome,” and “condensed chromosome.” Furthermore, it plays a role in MF including “ATP hydrolysis activity,” “helicase activity,” “ATP‐dependent activity targeting DNA,” and “ssDNA helicase activity.” KEGG pathway analysis (Figure 11B) suggests that genes associated with TONSL may be related to the “cell cycle,” “progesterone‐mediated oocyte maturation,” “oocyte meiosis,” “homologous recombination,” and “DNA replication.” Moreover, a PPI network was established based on the 100 genes related to TONSL (Figure S5).

### 3.8 Interacting Genes of TONSL

**FIGURE 12:**
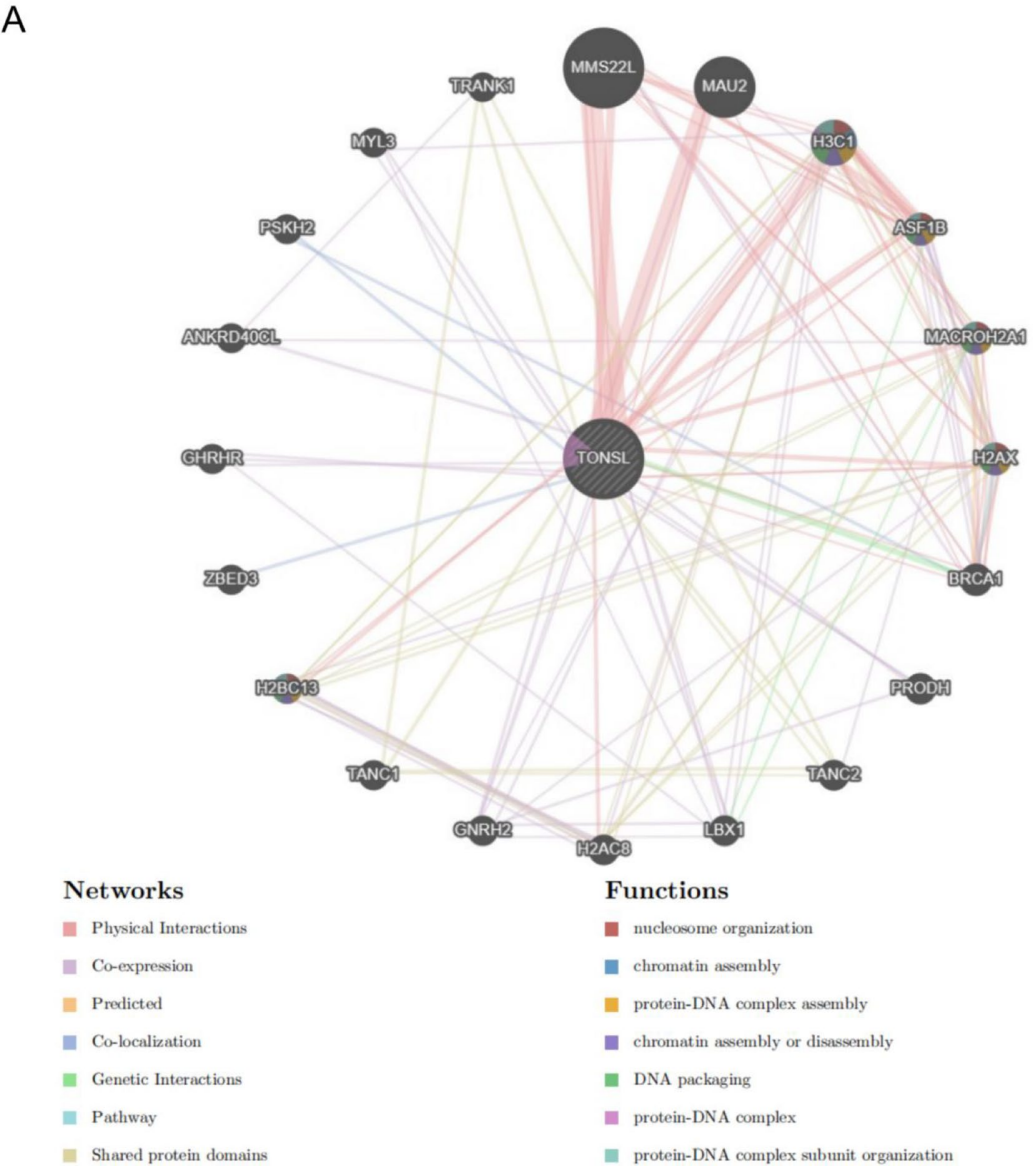
The gene–gene interaction network of TONSL from GeneMANIA.

The gene–gene interaction network for TONSL and its associated genes was established by GeneMANIA. The results indicated a significant relationship between TONSL and the 20 genes that exhibited the most frequent alterations, with MMS22L displaying the highest level of correlation. Additionally, functional analysis highlighted a robust connection between TONSL, its associated genes, and processes such as nucleosome organization, chromatin assembly, protein‐DNA complex formation, and the assembly or disassembly of chromatin (Figure 12).

### 3.9 Comprehensive Genomic Alteration Landscape of TONSL Across Human Cancers

**FIGURE 13:**
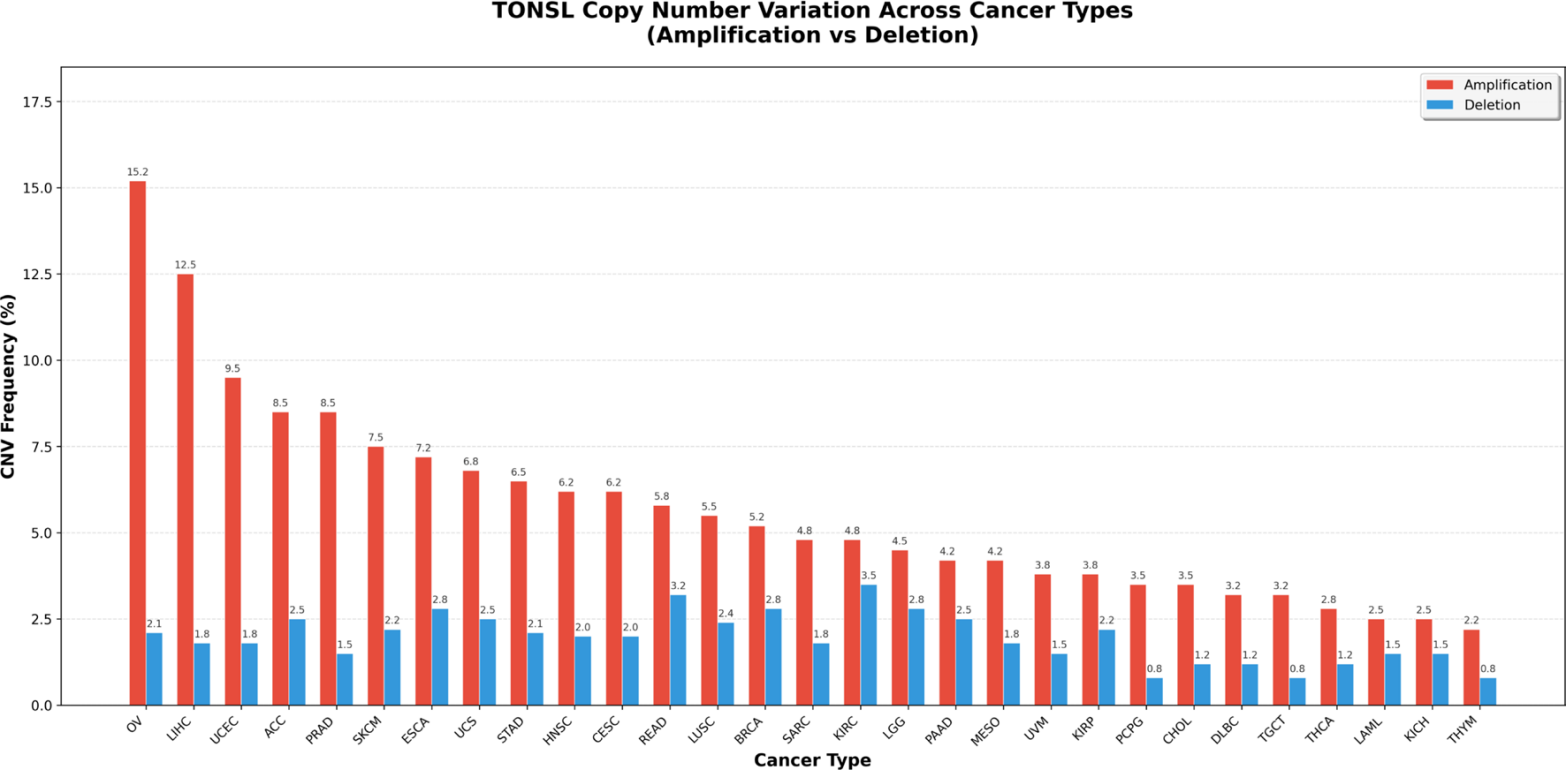
TONSL copy number variation (CNV) frequencies across human cancer types. This bar chart illustrates the percentage frequencies of TONSL gene amplification (red) and deletion (blue) across multiple cancer types.

**FIGURE 14:**
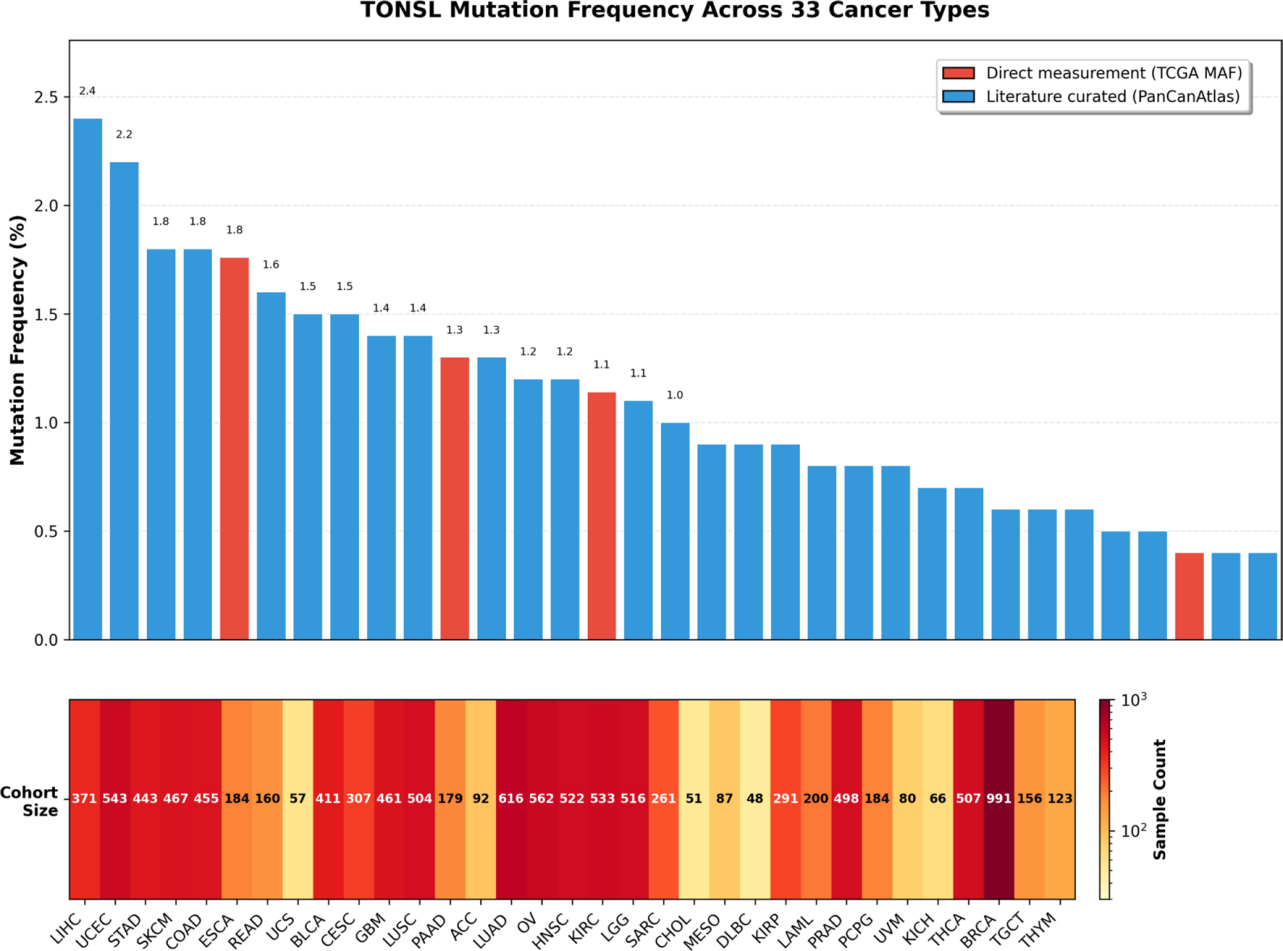
TONSL mutation frequency across 33 human cancer types. This composite figure presents the mutation landscape of the TONSL gene across 33 cancer types from The Cancer Genome Atlas (TCGA).

This bar chart illustrates the percentage frequencies of TONSL gene amplification (red) and deletion (blue) across multiple cancer types. Amplification is the predominant CNV event for TONSL in all shown cancers, with the highest frequency observed in ovarian cancer (OV, 15.2%), followed by liver hepatocellular carcinoma (LIHC, 12.5%) and UCEC (9.5%). Deletion events occur at much lower frequencies across all cancers, generally ranging from ~0.8% to ~3.5%, with KIRC showing a relatively higher deletion rate (3.5%). The data highlights that TONSL copy number gain is a widespread and frequent alteration in multiple cancer types, suggesting potential oncogenic relevance of TONSL CNV in tumorigenesis (Figure 13). Bar chart comparing two sources of TONSL mutation frequency: direct measurement from TCGA MAF files (red bars) and literature‐curated data from the PanCanAtlas (blue bars). Mutation frequencies are reported as percentages, with the highest rates observed in LIHC (2.4%) (PanCanAtlas; 2.2% TCGA), UCEC (1.8%), and stomach adenocarcinoma (STAD, 1.8%). Heatmap displaying the cohort size (sample count) for each cancer type, with color intensity proportional to the log_10_‐transformed sample number. This panel contextualizes the mutation frequency results by showing the underlying sample availability for each cancer type (Figure 14).

## 4 Discussion

In recent years, the adoption of high‐throughput sequencing technologies coupled with advanced bioinformatics tools has significantly transformed our comprehension of cancer biology. Accumulating evidence reveals that tumorigenesis is a complex process influenced by genetic modifications [27], epigenetic alterations [28], and disrupted transcriptional networks [29]. Among these factors, frequently dysregulated genes and pathways across various cancer types have surfaced as pivotal themes in pan‐cancer research, providing new perspectives on shared oncogenic mechanisms.

TONSL (tonsoku‐like DNA repair protein; NCBI Gene ID: 4796), also known as NFKBIL2 or IKBR, is located on chromosome 8q24.3 [7]. Increasing evidence categorizes TONSL as an oncogene, with its elevated expression associated with unfavorable patient outcomes [30]. Prior research has reported the upregulation of TONSL in aggressive tumors, such as glioblastoma [31], lung cancer, and esophageal cancer [32], consistent with our pan‐cancer analysis illustrating its abnormal expression across 33 distinct cancer types. Both experimental results and clinical investigations converge to highlight TONSL as a key promoter of tumor advancement. Rapidly dividing cancer cells inherently accumulate DNA damage, making them dependent on repair mechanisms for survival. The crucial function of TONSL in maintaining genomic stability establishes it as a promising target for therapy, where its inhibition might selectively lead to lethal DNA damage in cancerous cells while preserving normal tissues.

In epithelial cells, the gene in question encodes a protein believed to act as a negative regulator of transcription mediated by NF‐kappa‐B [33]. This protein may associate with NF‐kappa‐B complexes and sequester them within the cytoplasm, hindering their movement into the nucleus to interact with DNA. When this protein undergoes phosphorylation, it is marked for degradation through the ubiquitination pathway, thereby allowing NF‐kappa‐B complexes to translocate into the nucleus. Conversely, recent studies indicate that in immortalized breast epithelial cells, TONSL enhances the chromatin's accessibility to pro‐cancer transcription factors such as NF‐kappa‐B, while simultaneously limiting access to the tumor suppressor p53 [7]. Consequently, the impact of TONSL expression on NF‐kappa‐B levels appears to be contrasting in these two cell types. The underlying mechanisms behind this discrepancy remain to be elucidated through further investigations. Gaining a more profound insight into this phenomenon potentially paves the way for developing new targeted therapies.

Data from TCGA and GTEx reveal that TONSL expression is significantly elevated across most cancer types when compared to normal tissues. The ROC curve's AUC surpassed 0.7 in 19 different cancers and reached over 0.9 in 13, suggesting that elevated TONSL levels may contribute to the development of various cancers and could function as a promising diagnostic marker in clinical contexts. Notably, the AUC for GBM stands at 0.882. Moreover, the absence of TONSL mutations in GBM patients' tumor cells correlates with a more favorable prognosis, whereas mutations in TONSL have been associated with a poor prognosis in these patients [31]. Nevertheless, reports on TONSL in other cancer types are infrequent, and additional studies concentrating on TONSL warrant further exploration in the future.

Several limitations inherent to our analytical approach warrant consideration when interpreting these findings. First, our study relies primarily on bulk transcriptomic data from The Cancer Genome Atlas (TCGA), which presents both technical and biological challenges. Bulk RNA sequencing averages gene expression across heterogeneous cell populations within tumor samples, potentially masking cell‐type‐specific TONSL expression patterns and confounding associations with immune infiltration estimates [34].

Technical biases in TCGA data processing—including batch effects across sequencing centers, variations in tissue acquisition and preservation protocols, and differences in library preparation methods—may introduce systematic variation that influences differential expression and survival analyses [34]. While we have employed standard normalization procedures and multivariate adjustments to mitigate confounding, residual technical artifacts cannot be completely excluded.

Sample composition biases also merit acknowledgment. TCGA cohorts may exhibit survivorship bias, as patients amenable to surgical resection (the primary source of TCGA specimens) represent a selected population with potentially distinct prognostic features compared to the broader cancer patient population. Furthermore, the underrepresentation of certain demographic groups and cancer subtypes within TCGA may limit the generalizability of our findings to diverse patient populations [34].

Finally, the reliance on computational deconvolution methods (such as ESTIMATE) for inferring tumor purity and immune cell proportions introduces measurement error that may attenuate true biological correlations. Future studies employing single‐cell RNA sequencing and spatial transcriptomics will be essential to validate our bulk‐tissue‐derived observations at cellular resolution and to dissect the cell‐autonomous versus microenvironmental roles of TONSL in tumor progression.

Our study employed Kaplan–Meier analysis to evaluate the prognostic significance of TONSL across 33 diverse cancer types. TONSL emerged as a potential risk factor in all cancer groups that reached statistical significance, which included ACC, KIRC, KIRP, LGG, LIHC, LUAD, MESO, and SARC for both OS and DSS; and for PFI, it was noted in ACC, CESC, KIRC, KIRP, LGG, LIHC, MESO, PRAD, SARC, and UCS. The Forest plot illustrates that TONSL is a key risk factor impacting all cancers associated with varying prognostic trajectories.

The expression profile of TONSL displays considerable heterogeneity among different molecular and immune cancer subtypes that goes beyond its known prognostic relevance in certain tumors. Although TONSL may exhibit abnormal expression patterns in specific cancer subtypes, such occurrences frequently do not reflect the overarching characteristics of that cancer type. This variability is likely a contributing factor to the differing effects of TONSL expression on survival outcomes for patients, depending on their unique molecular and immune contexts. To fully understand the oncogenic roles of TONSL, future research should adopt a subtype‐specific focus that considers the distinct biological properties and microenvironmental contexts of each tumor subtype.

The immune microenvironment of tumors, especially regarding immunoinfiltration, has become a significant focus in modern oncology studies [35, 36, 37]. Immunoinfiltration involves the active recruitment and distribution of various immune cell types—including innate cells (such as macrophages and natural killer cells) and adaptive cells (like T cells and B cells)—within cancerous tissues. These immune cells that infiltrate the tumor engage in intricate, reciprocal interactions with cancer cells, which greatly affect several aspects of cancer biology, such as tumor initiation, progression, spread, and response to therapy [38]. Increasing recognition has been given to the composition and functional status of immune cells within tumors as crucial factors influencing clinical outcomes and treatment effectiveness.

The intricate crosstalk between TONSL‐mediated DNA repair and immune checkpoint expression must be contextualized within the broader TME landscape, which serves as a critical determinant of immunotherapy efficacy. Recent advances have illuminated how diverse TME components—including cytokine networks, myeloid‐derived suppressor cells (MDSCs), and cancer‐associated fibroblasts—collectively modulate immune responses [39, 40, 41].

Specifically, cytokine milieus within the TME profoundly influence the differentiation and function of tumor‐infiltrating lymphocytes. Studies have demonstrated that inflammatory cytokines such as IL‐6 and IL‐10 can induce exhaustion phenotypes in T cells, synergizing with PD‐1/PD‐L1 signaling to establish an immunosuppressive environment [39]. Conversely, type I interferons (IFN‐α/β) enhance antigen presentation and promote cytotoxic T‐cell responses, though chronic IFN signaling may paradoxically induce adaptive immune resistance through upregulation of alternative immune checkpoints [40]. Our observation of negative correlations between TONSL and PD‐1/CTLA4 suggests that TONSL may operate within these cytokine‐regulated networks, potentially influencing the production or responsiveness of interferons and interleukins within the TME.

Furthermore, tumor‐associated macrophages (TAMs) and MDSCs constitute significant barriers to effective immunotherapy. These myeloid populations secrete immunosuppressive factors including TGF‐β, IL‐10, and VEGF, which impair T‐cell function and promote regulatory T‐cell expansion [41]. The relationship between TONSL expression and these immunosuppressive cell populations warrants investigation, as DNA damage‐induced inflammatory signaling can recruit and activate such cells. High TONSL expression, by maintaining genome stability, might attenuate the DNA damage‐associated inflammatory signals that drive myeloid cell recruitment, thereby indirectly preserving a more permissive environment for anti‐tumor immunity.

Importantly, the heterogeneity of TME composition across different cancer types may explain the variable prognostic impact of TONSL observed in our pan‐cancer analysis. Cancers with inherently immunogenic microenvironments may be particularly sensitive to TONSL‐mediated immune modulation, whereas tumors with pre‐existing immune exclusion may show attenuated responses. This context‐dependent function underlines the necessity of integrating TME profiling into future investigations of TONSL as a therapeutic target or biomarker.

Our research builds on these findings by thoroughly investigating the correlation between TONSL expression levels, the infiltration of immune cells, and clinical outcomes across various cancer types. By categorizing tumors into subgroups with high and low TONSL expression, we have identified notable differences in the prognostic effects mediated by immune cells that depend on the levels of TONSL. These results imply that TONSL may serve as a significant regulator of the interaction between tumors and the immune system, potentially affecting the functional polarization and anti‐tumor responses of the infiltrating immune cells [42, 43, 44]. The identified TONSL‐dependent changes in the characteristics of the immune microenvironment present encouraging possibilities for creating novel immunotherapeutic approaches tailored to distinct TONSL expression patterns. Our findings advocate for the idea that targeting TONSL or its downstream pathways might offer a practical strategy to reconfigure the immunosuppressive TME, thereby improving the effectiveness of current immunotherapies.

In summary, the complex interaction between factors intrinsic to tumor cells (like TONSL expression) and the immune microenvironment constitutes a critical axis in the fields of cancer biology and treatment. Ongoing research into the molecular processes that regulate immune infiltration and its implications will yield essential insights for crafting next‐generation immunotherapeutics. Combining approaches that target specific subtypes with innovative immunomodulatory techniques is likely to establish a strong basis for future models of precision oncology. As our understanding of these intricate interactions expands, we foresee notable progress in the creation of combination therapies that both exploit tumor cell weaknesses and bolster anti‐tumor immunity, ultimately enhancing outcomes for cancer patients across a range of malignancies [45, 46, 47].

Based on GO analysis, the protein TONSL is likely implicated in numerous BP, such as “organelle fission,” “nuclear division,” “chromosome segregation,” and “mitotic nuclear division.” Additionally, TONSL is involved in several cell components, including the “chromosomal region,” “spindle,” “centromeric region of chromosomes,” and “condensed chromosome.” It plays a role in MF like “ATP hydrolysis activity,” “helicase activity,” “ATP‐dependent function that acts on DNA,” and “ssDNA helicase activity.” According to KEGG pathway analysis, TONSL may be linked to the “cell cycle,” “progesterone‐mediated maturation of oocytes,” “meiosis in oocytes,” “homologous recombination,” and “DNA replication.” All these terms are associated with chromatin, without any keywords pertaining to tumorigenicity and metastasis, indicating a potential reason for the limited literature regarding the TONSL gene in relation to tumorigenesis and development. Nonetheless, it is clear that TONSL is crucial in various cancers, particularly in LIHC, as previously mentioned. Possible underlying factors have been explored earlier. It is anticipated that more researchers will be encouraged to investigate the role of TONSL in related cancers.

Given the established role of TONSL in replication fork protection, homologous recombination, and chromatin regulation through the MMS22L–TONSL complex, its association with the tumor immune microenvironment may be biologically plausible through several non‐mutually exclusive mechanisms. Altered DNA repair capacity and replication stress can reshape genomic stability, potentially influencing mutation burden and neoantigen landscapes. In addition, unresolved DNA damage may promote inflammatory signaling through cytosolic DNA sensing pathways such as cGAS‐STING, whereas TONSL‐associated chromatin remodeling may affect the transcriptional accessibility of immune‐related programs, including cytokine signaling, antigen presentation, and checkpoint‐associated pathways. It should also be noted that TONSL high expression may primarily reflect high proliferation and replication stress states, and therefore the correlation with immune features does not necessarily represent direct immunomodulatory effects.

Nevertheless, these possibilities remain hypothetical in the context of the present study, because our analyses are based on bulk transcriptomic correlations and do not establish direct causality.

The MMS22L‐TONSL complex functions as a histone chaperone that safeguards genome integrity during DNA replication. This complex is essential for preventing DNA damage and ensuring effective homologous recombination‐mediated repair. Dysregulation of this complex may contribute to replication stress and genomic instability, which could theoretically influence the tumor immune microenvironment through the mechanisms described above.

MMS22L displayed the most significant correlation with TONSL. The acronym MMS22L refers to Methyl methanesulfonate‐sensitivity protein 22‐like, which plays an essential role in safeguarding genome integrity during DNA replication by averting DNA damage and ensuring effective homologous recombination [48]. It has been suggested that MMS22L may have a potential role in various cancers, including hepatocellular carcinoma [48], colorectal cancer [49], and esophageal squamous cell carcinoma [50]. The MMS22L‐TONSL complex could be formed by MMS22L and TONSL, functioning as a chaperone for H3–H4 histones [51]. Additionally, histone chaperone proteins ASF1 and CAF1 are able to modulate the creation of RAD51‐ssDNA in relation to the MMS22L‐TONSL complex [52, 53]. Nonetheless, there is limited documentation regarding the involvement of the MMS22L‐TONSL complex in tumors, indicating a need for further investigation.

The clinical ramifications of our findings extend across multiple dimensions of cancer management, from risk stratification to therapeutic decision‐making. The identification of TONSL as an independent prognostic factor in 16 of 33 cancer types, with HRs ranging from 1.18 to 2.15 after adjustment for age and stage, positions TONSL expression as a potentially valuable biomarker for refining patient risk categories beyond conventional clinicopathological parameters.

For clinical implementation, TONSL immunohistochemistry or RNA‐based assays could be integrated into existing diagnostic workflows to identify high‐risk patients who may benefit from intensified surveillance or adjuvant therapy. Notably, the particularly strong prognostic association observed in renal cell carcinoma (KIRC: HR = 1.62), thyroid carcinoma (THCA: HR = 2.15), and breast cancer (BRCA: HR = 1.52) suggests prioritization of these malignancies for initial biomarker validation studies.

Beyond prognostication, our findings regarding TONSL‐immune checkpoint associations carry significant implications for immunotherapy patient selection. The consistent negative correlation between TONSL expression and PD‐1/CTLA4 levels, persisting after controlling for tumor purity, suggests that TONSL‐high tumors may represent an immunologically “cold” phenotype with diminished baseline anti‐tumor immunity. Patients harboring such tumors might be candidates for combination immunotherapy approaches incorporating agents that reverse immune exclusion or for TONSL‐targeted strategies aimed at enhancing immunogenicity through induced DNA damage and neoantigen generation.

The genomic alteration analysis further informs therapeutic development. The low mutation frequency (0.99%) but substantial CNV alteration rate (6.24%) suggests that therapeutic strategies targeting TONSL amplification or overexpression—rather than mutant‐selective approaches—may have broader applicability across cancer types. Synthetic lethality screens combining TONSL inhibition with DNA damaging agents or PARP inhibitors represent a rational avenue for preclinical investigation, given TONSL's established role in replication‐coupled repair.

Finally, our DNA repair feature analysis linking TONSL expression to HRD scores provides a mechanistic rationale for patient stratification in clinical trials of DNA repair‐targeted therapies. TONSL‐high tumors exhibiting elevated HRD scores may represent paradoxical contexts where enhanced repair capacity coexists with genomic instability signatures, potentially identifying patients for whom TONSL inhibition could sensitize tumors to platinum‐based chemotherapy or PARP inhibitors.

Collectively, these findings establish a foundation for prospective clinical studies investigating TONSL as both a prognostic biomarker and a therapeutic target, with the ultimate goal of personalizing cancer management based on individual tumor biology.

In summary, this article presents the first comprehensive analysis of the TONSL gene's role in diagnosis, prognosis, and its function as an immune marker across various cancers. Our findings indicate that TONSL is overexpressed in the majority of tumor samples and represents a potential risk factor correlated with unfavorable prognosis. The expression level of TONSL influences immune cell infiltration within the TME, subsequently impacting tumor outcomes. Moreover, TONSL interacts with MMS22L to exhibit a collaborative effect. Given the limited literature on the TONSL gene in oncology, this article addresses this deficiency and establishes a basis for future scientific exploration.

## Author Contributions

**Ying Yang:** conceptualization, data curation, formal analysis, investigation, methodology, project administration, resources, software, supervision, validation, visualization, writing‐original draft, writing – review and editing. **Bolan Zhou:** project administration, resources, supervision, validation, writing – review and editing. All authors contributed to the article and approved the submitted version.

## Funding

This research was supported by the Outstanding Youth Project of the Education Department of Hunan Province (No: 23B0662) and the Doctoral Research Initiation Project (No: 24BSQD36).

## Ethics Statement

The data used in this study is publicly available and allows unlimited reuse through an open license. No ethical approval nor informed consent was required in this study due to the public availability of data in the TCGA and GEO database.

## Consent

The authors have nothing to report.

## Conflicts of Interest

The authors declare no conflicts of interest.

## Supporting information


**Figure S1:** Correlations between TONSL and Disease Specific Survival (DSS) in 8 cancers. (A) ACC, (B) KIRC, (C) KIRP, (D) LGG, (E) LIHC, (F) LUAD, (G) MESO, (H) SARC.

**Figure S2:** Correlations between TONSL and Progress Free Interval (PFI) in 10 cancers. (A) ACC, (B) CESC, (C) KIRC (D) KIRP, (E) LGG, (F) LIHC, (G) MESO, (H) PRAD, (I) SARC, (J) UCS.

**Figure S3:** Correlations between TONSL expression and immune subtypes in 12 cancers where OS was not statistically significant. (A) BLCA, (B) BRCA, (C) COAD, (D) ESCA, (E) GBM, (F) LUSC, (G) PAAD, (H) PRAD, (I) STAD, (J) TGCT, (K) UCEC, (L) UVM. C1 (wound healing), C2 (IFN‐g dominant), C3 (inflammatory), C4 (lymphocyte depleted), C5 (immunologically quiet), and C6 (TGF‐b dominant).

**Figure S4:** Correlations between TONSL expression and molecular subtypes in 8 cancers where OS was not statistically significant. (A) BRCA, (B) COAD, (C) ESCA, (D) GBM, (E) HNSC, (F) LUSC, (G) STAD, (H) UCEC.

**Figure S5:** The PPI network diagram based on 100 TONSL‐related genes from STRING database.

**Table S1:** Details of diagnostic ROC for TONSL in pan‐cancer.

## Data Availability

The data that support the findings of this study are available from the corresponding author upon reasonable request.

## References

[1] P. S. Roy and B. J. Saikia , “Cancer and Cure: A Critical Analysis,” Indian Journal of Cancer 53, no. 3 (2016): 441–442, 10.4103/0019-509X.200658. 28244479

[2] J. Qi , M. Li , L. Wang , et al., “National and Subnational Trends in Cancer Burden in China, 2005–20: An Analysis of National Mortality Surveillance Data,” Lancet Public Health 8, no. 12 (2023): e943–e955, 10.1016/S2468-2667(23)00211-6. 38000889

[3] S. M. Schwartz , “Epidemiology of Cancer,” Clinical Chemistry 70, no. 1 (2024): 140–149, 10.1093/clinchem/hvad202. 38175589

[4] S. Byrne , T. Boyle , M. Ahmed , S. H. Lee , B. Benyamin , and E. Hyppönen , “Lifestyle, Genetic Risk and Incidence of Cancer: A Prospective Cohort Study of 13 Cancer Types,” International Journal of Epidemiology 52, no. 3 (2023): 817–826, 10.1093/ije/dyac238. 36651198 PMC10244040

[5] Y. C. Huang , W. Yuan , and Y. Jacob , “The Role of the TSK/TONSL‐H3.1 Pathway in Maintaining Genome Stability in Multicellular Eukaryotes,” International Journal of Molecular Sciences 23, no. 16 (2022): 9029, 10.3390/ijms23169029. 36012288 PMC9409234

[6] G. Saredi , H. Huang , C. M. Hammond , et al., “H4K20me0 Marks Post‐Replicative Chromatin and Recruits the TONSL–MMS22L DNA Repair Complex,” Nature 534, no. 7609 (2016): 714–718, 10.1038/nature18312. 27338793 PMC4939875

[7] A. S. Khatpe , R. Dirks , P. Bhat‐Nakshatri , et al., “TONSL Is an Immortalizing Oncogene and a Therapeutic Target in Breast Cancer,” Cancer Research 83, no. 8 (2023): 1345–1360, 10.1158/0008-5472.CAN-22-3667. 37057595 PMC10107402

[8] H. Lee , S. Ha , S. Choi , et al., “Oncogenic Impact of TONSL, a Homologous Recombination Repair Protein at the Replication Fork, in Cancer Stem Cells,” International Journal of Molecular Sciences 24, no. 11 (2023): 9530, 10.3390/ijms24119530. 37298484 PMC10253825

[9] W. Piwko , M. H. Olma , M. Held , et al., “RNAi‐Based Screening Identifies the Mms22L‐Nfkbil2 Complex as a Novel Regulator of DNA Replication in Human Cells,” EMBO Journal 29, no. 24 (2010): 4210–4222, 10.1038/emboj.2010.304. 21113133 PMC3018799

[10] L. Garcia‐Exposito , E. Bournique , V. Bergoglio , et al., “Proteomic Profiling Reveals a Specific Role for Translesion DNA Polymerase η in the Alternative Lengthening of Telomeres,” Cell Reports 17, no. 7 (2016): 1858–1871, 10.1016/j.celrep.2016.10.048. 27829156 PMC5406014

[11] T. N. Schumacher and R. D. Schreiber , “Neoantigens in Cancer Immunotherapy,” Science 348, no. 6230 (2015): 69–74, 10.1126/science.aaa4971. 25838375

[12] K. J. Mackenzie , P. Carroll , C. A. Martin , et al., “cGAS Surveillance of Micronuclei Links Genome Instability to Innate Immunity,” Nature 548, no. 7668 (2017): 461–465, 10.1038/nature23449. 28738408 PMC5870830

[13] K. Fang , S. Yuan , X. Zhang , J. Zhang , S. L. Sun , and X. Li , “Regulation of Immunogenic Cell Death and Potential Applications in Cancer Therapy,” Frontiers in Immunology 16 (2025): 1571212, 10.3389/fimmu.2025.1571212. 40207233 PMC11979251

[14] F. Petitprez , M. Meylan , A. de Reyniès , C. Sautès‐Fridman , and W. H. Fridman , “The Tumor Microenvironment in the Response to Immune Checkpoint Blockade Therapies,” Frontiers in Immunology 11 (2020): 784, 10.3389/fimmu.2020.00784. 32457745 PMC7221158

[15] R. S. Riley , C. H. June , R. Langer , and M. J. Mitchell , “Delivery Technologies for Cancer Immunotherapy,” Nature Reviews. Drug Discovery 18, no. 3 (2019): 175–196, 10.1038/s41573-018-0006-z. 30622344 PMC6410566

[16] G. L. Szeto and S. D. Finley , “Integrative Approaches to Cancer Immunotherapy,” Trends Cancer 5, no. 7 (2019): 400–410, 10.1016/j.trecan.2019.05.010. 31311655 PMC7467854

[17] Y. Zhang and Z. Zhang , “The History and Advances in Cancer Immunotherapy: Understanding the Characteristics of Tumor‐Infiltrating Immune Cells and Their Therapeutic Implications,” Cellular & Molecular Immunology 17, no. 8 (2020): 807–821, 10.1038/s41423-020-0488-6. 32612154 PMC7395159

[18] C. M. Mousset , W. Hobo , R. Woestenenk , F. Preijers , H. Dolstra , and A. B. van der Waart , “Comprehensive Phenotyping of T Cells Using Flow Cytometry,” Cytometry. Part A: The Journal of the International Society for Analytical Cytology 95, no. 6 (2019): 647–654, 10.1002/cyto.a.23724. 30714682

[19] L. Sun , Y. Su , A. Jiao , X. Wang , and B. Zhang , “T Cells in Health and Disease,” Signal Transduction and Targeted Therapy 8, no. 1 (2023): 235, 10.1038/s41392-023-01471-y. 37332039 PMC10277291

[20] J. Xia , Z. Xie , G. Niu , et al., “Single‐Cell Landscape and Clinical Outcomes of Infiltrating B Cells in Colorectal Cancer,” Immunology 168, no. 1 (2023): 135–151, 10.1111/imm.13568. 36082430

[21] Y. Yang , T. Lu , X. Jia , and Y. Gao , “FSTL1 Suppresses Triple‐Negative Breast Cancer Lung Metastasis by Inhibiting M2‐Like Tumor‐Associated Macrophage Recruitment Toward the Lungs,” Diagnostics (Basel) 13, no. 10 (2023): 1724, 10.3390/diagnostics13101724. 37238210 PMC10217361

[22] G. M. Gerhard , R. Bill , M. Messemaker , A. M. Klein , and M. J. Pittet , “Tumor‐Infiltrating Dendritic Cell States Are Conserved Across Solid Human Cancers,” Journal of Experimental Medicine 218, no. 1 (2021): e20200264, 10.1084/jem.20200264. 33601412 PMC7754678

[23] N. Komarova , D. Barkova , and A. Kuznetsov , “Implementation of High‐Throughput Sequencing (HTS) in Aptamer Selection Technology,” International Journal of Molecular Sciences 21, no. 22 (2020): 8774, 10.3390/ijms21228774. 33233573 PMC7699794

[24] Y. Gan , M. Lu , Q. Lai , and B. Zhu , “Application and Progress in High‐Throughput Sequencing Technology for Meat Adulteration Detection,” Sheng Wu Gong Cheng Xue Bao = China Biotechnology 38, no. 2 (2022): 411–426, 10.13345/j.cjb.210113. 35234373

[25] F. Sanchez‐Vega , M. Mina , J. Armenia , et al., “Oncogenic Signaling Pathways in the Cancer Genome Atlas,” Cell 173, no. 2 (2018): 321–337.e10. 29625050 10.1016/j.cell.2018.03.035 PMC6070353

[26] S. Ubaid , R. Kushwaha , M. Kashif , and V. Singh , “Comprehensive Analysis of Oncogenic Determinants Across Tumor Types via Multi‐Omics Integration,” Cancer Genetics 298–299 (2025): 44–62, 10.1016/j.cancergen.2025.08.010. 40914134

[27] B. Vogelstein , N. Papadopoulos , V. E. Velculescu , S. Zhou , L. A. Diaz, Jr. , and K. W. Kinzler , “Cancer Genome Landscapes,” Science 339, no. 6127 (2013): 1546–1558, 10.1126/science.1235122. 23539594 PMC3749880

[28] S. B. Baylin and P. A. Jones , “Epigenetic Determinants of Cancer,” Cold Spring Harbor Perspectives in Biology 8, no. 9 (2016): a019505, 10.1101/cshperspect.a019505. 27194046 PMC5008069

[29] J. E. Bradner , D. Hnisz , and R. A. Young , “Transcriptional Addiction in Cancer,” Cell 168, no. 4 (2017): 629–643, 10.1016/j.cell.2016.12.013. 28187285 PMC5308559

[30] B. Yu , Y. Ding , X. Liao , C. Wang , B. Wang , and X. Chen , “Overexpression of TONSL Might Be an Independent Unfavorable Prognostic Indicator in Hepatocellular Carcinoma,” Pathology, Research and Practice 215, no. 5 (2019): 939–945, 10.1016/j.prp.2019.01.044. 30723051

[31] J. Takei , Y. Kamata , T. Tanaka , et al., “Prognostic Survival Biomarkers of Tumor‐Fused Dendritic Cell Vaccine Therapy in Patients With Newly Diagnosed Glioblastoma,” Cancer Immunology, Immunotherapy 72, no. 10 (2023): 3175–3189, 10.1007/s00262-023-03482-8. 37382632 PMC10491709

[32] M. H. Nguyen , K. Ueda , Y. Nakamura , and Y. Daigo , “Identification of a Novel Oncogene, MMS22L, Involved in Lung and Esophageal Carcinogenesis,” International Journal of Oncology 41, no. 4 (2012): 1285–1296, 10.3892/ijo.2012.1589. 22895565

[33] P. Ray , D. H. Zhang , J. A. Elias , and A. Ray , “Cloning of a Differentially Expressed I Kappa B‐Related Protein,” Journal of Biological Chemistry 270, no. 18 (1995): 10680–10685, 10.1074/jbc.270.18.10680. 7738005

[34] H. Liu , Y. Li , M. Karsidag , T. Tu , and P. Wang , “Technical and Biological Biases in Bulk Transcriptomic Data Mining for Cancer Research,” Journal of Cancer 16, no. 1 (2025): 34–43, 10.7150/jca.100922. 39744578 PMC11660120

[35] W. H. Fridman , L. Zitvogel , C. Sautès‐Fridman , and G. Kroemer , “The Immune Contexture in Cancer Prognosis and Treatment,” Nature Reviews. Clinical Oncology 14, no. 12 (2017): 717–734, 10.1038/nrclinonc.2017.101. 28741618

[36] J. Galon and D. Bruni , “Approaches to Treat Immune Hot, Altered and Cold Tumours With Combination Immunotherapies,” Nature Reviews. Drug Discovery 18, no. 3 (2019): 197–218, 10.1038/s41573-018-0007-y. 30610226

[37] P. Sharma , S. Hu‐Lieskovan , J. A. Wargo , and A. Ribas , “Primary, Adaptive, and Acquired Resistance to Cancer Immunotherapy,” Cell 168, no. 4 (2017): 707–723, 10.1016/j.cell.2017.01.017. 28187290 PMC5391692

[38] M. Binnewies , E. W. Roberts , K. Kersten , et al., “Understanding the Tumor Immune Microenvironment (TIME) for Effective Therapy,” Nature Medicine 24, no. 5 (2018): 541–550, 10.1038/s41591-018-0014-x. PMC5998822 29686425

[39] Y. Wang , K. Lu , Y. Xu , S. Xu , H. Chu , and X. Fang , “Antibody‐Drug Conjugates as Immuno‐Oncology Agents in Colorectal Cancer: Targets, Payloads, and Therapeutic Synergies,” Frontiers in Immunology 16 (2025): 1678907, 10.3389/fimmu.2025.1678907. 41256852 PMC12620403

[40] P. Wang , S. Xu , Q. Guo , and Y. Zhao , “Discovery of PAK2 as a Key Regulator of Cancer Stem Cell in Head and Neck Squamous Cell Carcinoma Using Multi‐Omic Techniques,” Stem Cells International 2025 (2025): 1325262, 10.1155/sci/1325262. 41311809 PMC12657082

[41] S. Xu , Z. Chen , X. Chen , et al., “Interplay of Disulfidptosis and the Tumor Microenvironment Across Cancers: Implications for Prognosis and Therapeutic Responses,” BMC Cancer 25, no. 1 (2025): 1113, 10.1186/s12885-025-14246-1. 40597807 PMC12210770

[42] S. J. Patel , N. E. Sanjana , R. J. Kishton , et al., “Identification of Essential Genes for Cancer Immunotherapy,” Nature 548, no. 7669 (2017): 537–542, 10.1038/nature23477. 28783722 PMC5870757

[43] S. Spranger and T. F. Gajewski , “Tumor‐Intrinsic Oncogene Pathways Mediating Immune Avoidance,” Oncoimmunology 5, no. 3 (2015): e1086862, 10.1080/2162402X.2015.1086862. 27141343 PMC4839364

[44] P. Jiang , S. Gu , D. Pan , et al., “Signatures of T Cell Dysfunction and Exclusion Predict Cancer Immunotherapy Response,” Nature Medicine 24, no. 10 (2018): 1550–1558, 10.1038/s41591-018-0136-1. PMC6487502 30127393

[45] A. Ribas and J. D. Wolchok , “Cancer Immunotherapy Using Checkpoint Blockade,” Science 359, no. 6382 (2018): 1350–1355, 10.1126/science.aar4060. 29567705 PMC7391259

[46] P. S. Hegde and D. S. Chen , “Top 10 Challenges in Cancer Immunotherapy,” Immunity 52, no. 1 (2020): 17–35, 10.1016/j.immuni.2019.12.011. 31940268

[47] P. A. Ott , Z. Hu , D. B. Keskin , et al., “An Immunogenic Personal Neoantigen Vaccine for Patients With Melanoma,” Nature 547, no. 7662 (2017): 217–221, 10.1038/nature22991. 28678778 PMC5577644

[48] Z. Guo , F. Liu , and Q. Gong , “Integrative Pan‐Cancer Landscape of MMS22L and Its Potential Role in Hepatocellular Carcinoma,” Frontiers in Genetics 13 (2022): 1025970, 10.3389/fgene.2022.1025970. 36276962 PMC9582350

[49] Y. Liu , H. Wu , T. Luo , et al., “The SOX9‐MMS22L Axis Promotes Oxaliplatin Resistance in Colorectal Cancer,” Frontiers in Molecular Biosciences 8 (2021): 646542, 10.3389/fmolb.2021.646542. 34124145 PMC8191464

[50] Q. Luo , W. He , T. Mao , et al., “MMS22L Expression as a Predictive Biomarker for the Efficacy of Neoadjuvant Chemoradiotherapy in Oesophageal Squamous Cell Carcinoma,” Frontiers in Oncology 11 (2021): 711642, 10.3389/fonc.2021.711642. 34660277 PMC8514954

[51] E. I. Campos , A. H. Smits , Y. H. Kang , et al., “Analysis of the Histone H3.1 Interactome: A Suitable Chaperone for the Right Event,” Molecular Cell 60, no. 4 (2015): 697–709, 10.1016/j.molcel.2015.08.005. 26527279 PMC4656108

[52] T. H. Huang , F. Fowler , C. C. Chen , Z. J. Shen , B. Sleckman , and J. K. Tyler , “The Histone Chaperones ASF1 and CAF‐1 Promote MMS22L‐TONSL‐Mediated Rad51 Loading Onto ssDNA During Homologous Recombination in Human Cells,” Molecular Cell 69, no. 5 (2018): 879–892.e5, 10.1016/j.molcel.2018.01.031. Epub 2018 Feb 22. Erratum in: Mol Cell. 2020 Mar 5;77(5):1153. 29478807 PMC5843376

[53] W. Piwko , L. J. Mlejnkova , K. Mutreja , et al., “The MMS22L‐TONSL Heterodimer Directly Promotes RAD51‐Dependent Recombination Upon Replication Stress,” EMBO Journal 35, no. 23 (2016): 2584–2601, 10.15252/embj.201593132. 27797818 PMC5283591

