# Supplementary figures and images for: Comprehensive Bioinformatic Analysis of TONSL Expression in Pan‐Cancer
Source: Cancer Rep (Hoboken). 2026 Apr 19;9(4):e70551. doi: 10.1002/cnr2.70551 (PMC13092426; doi:10.1002/cnr2.70551)

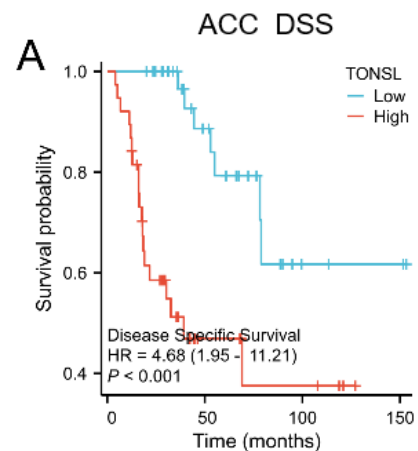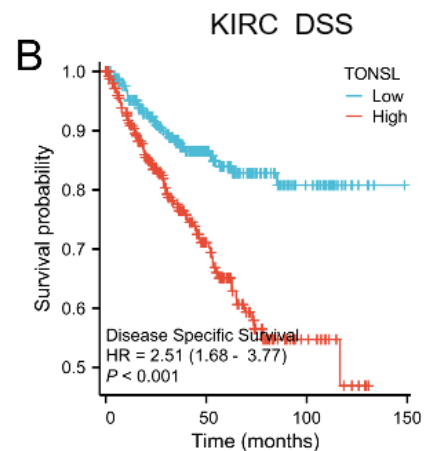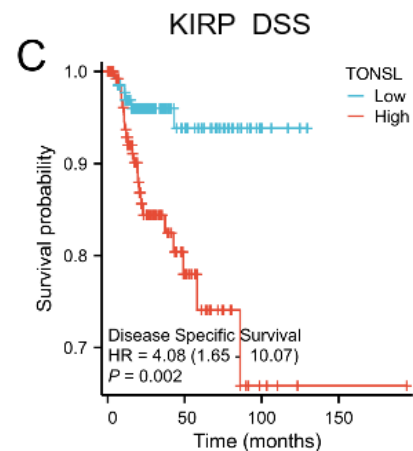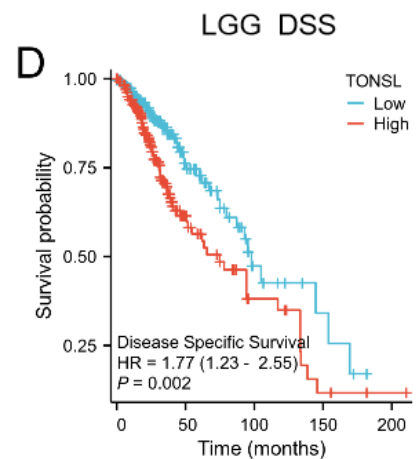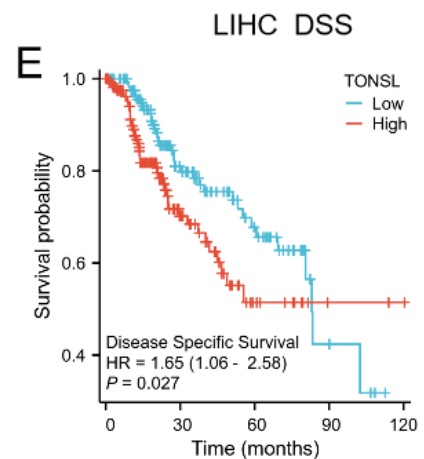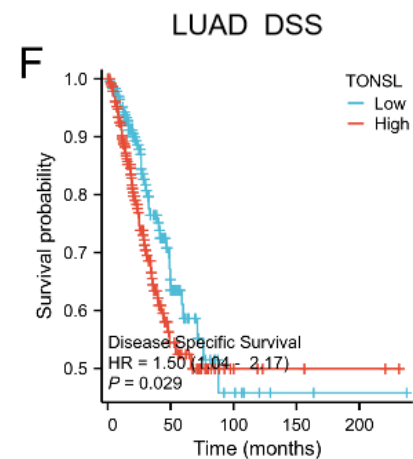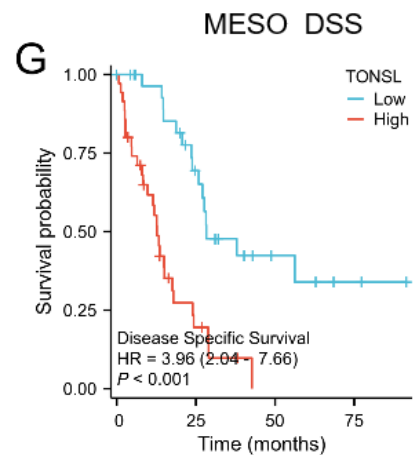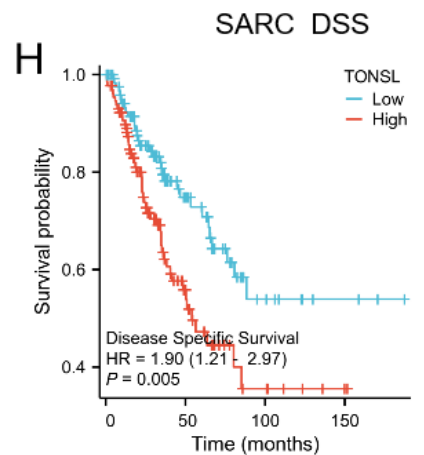

Supplement: Supplementary file 1 — Figure S1: Correlations between TONSL and Disease Specific Survival (DSS) in 8 cancers. (A) ACC, (B) KIRC, (C) KIRP, (D) LGG, (E) LIHC, (F) LUAD, (G) MESO, (H) SARC. [file CNR2-9-e70551-s004.pdf]

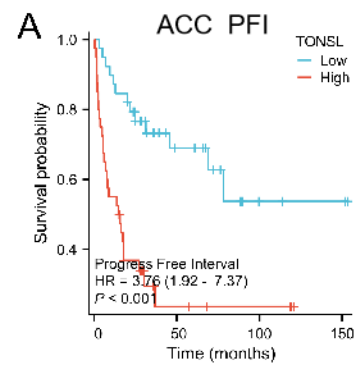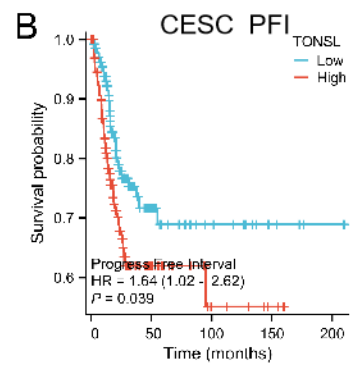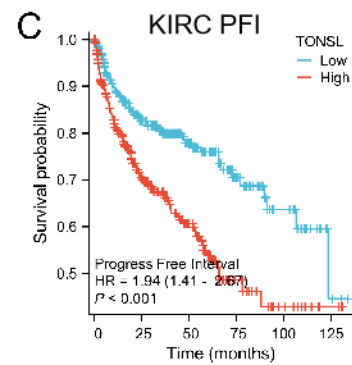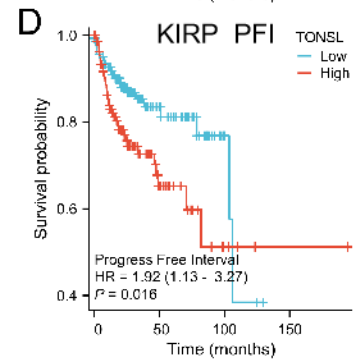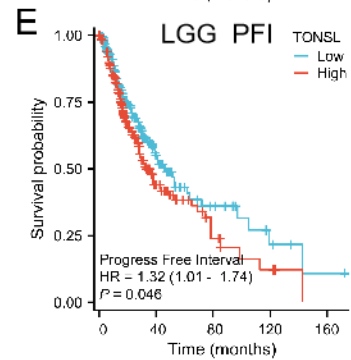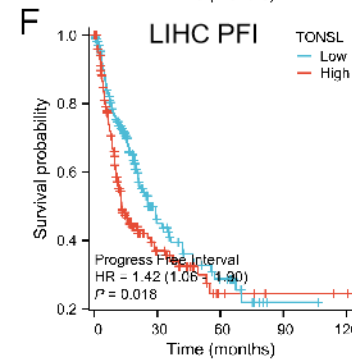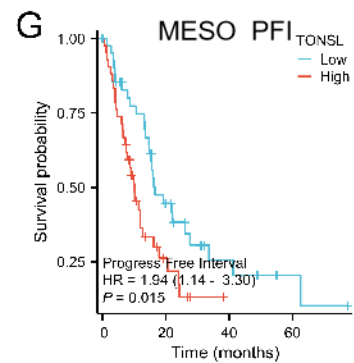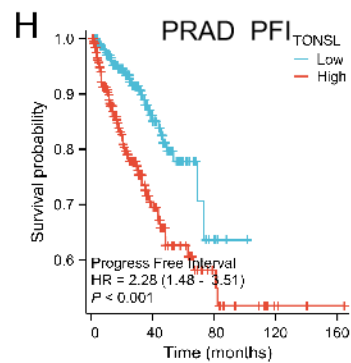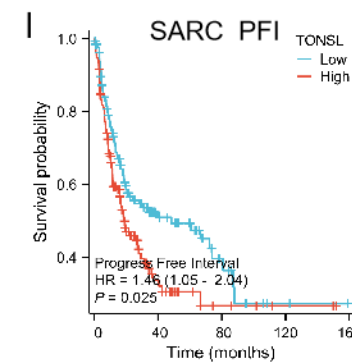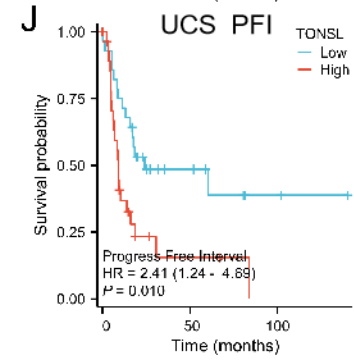

Supplement: Supplementary file 2 — Figure S2: Correlations between TONSL and Progress Free Interval (PFI) in 10 cancers. (A) ACC, (B) CESC, (C) KIRC (D) KIRP, (E) LGG, (F) LIHC, (G) MESO, (H) PRAD, (I) SARC, (J) UCS. [file CNR2-9-e70551-s002.pdf]

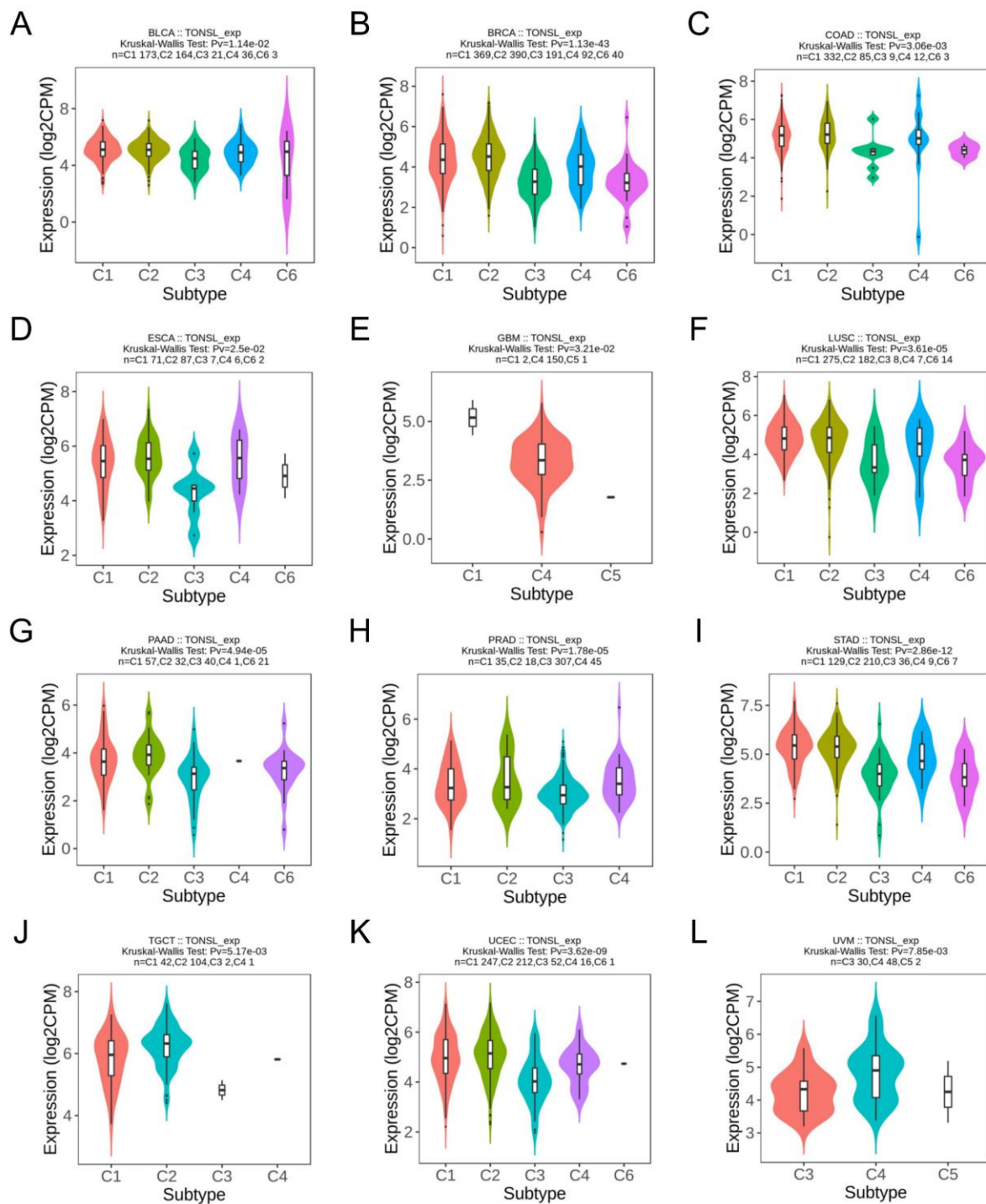

Supplement: Supplementary file 3 — Figure S3: Correlations between TONSL expression and immune subtypes in 12 cancers where OS was not statistically significant. (A) BLCA, (B) BRCA, (C) COAD, (D) ESCA, (E) GBM, (F) LUSC, (G) PAAD, (H) PRAD, (I) STAD, (J) TGCT, (K) UCEC, (L) UVM. C1 (wound healing), C2 (IFN‐g dominant), C3 (inflammatory), C4 (lymphocyte depleted), C5 (immunologically quiet), and C6 (TGF‐b dominant). [file CNR2-9-e70551-s006.pdf]

A

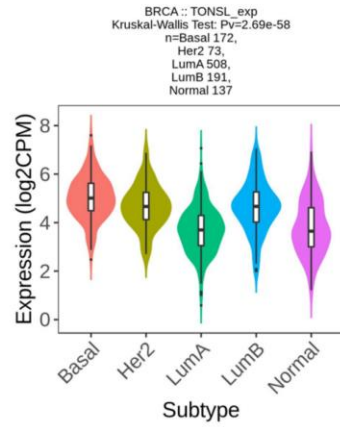

B

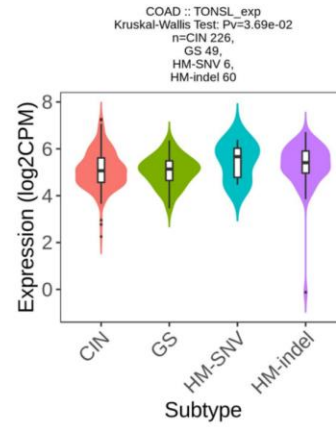

C

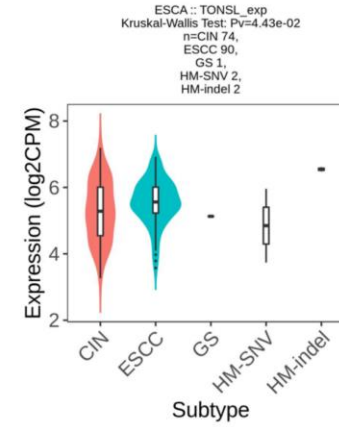

D

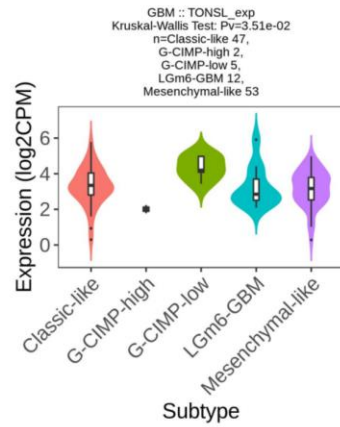

E

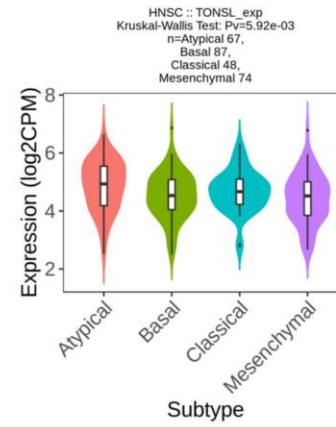

F

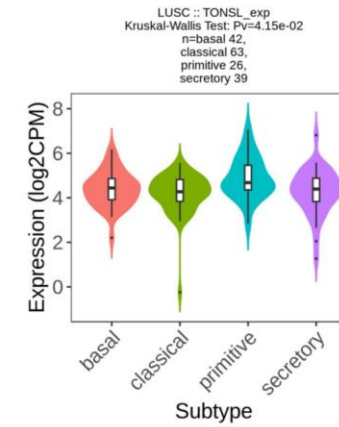

G

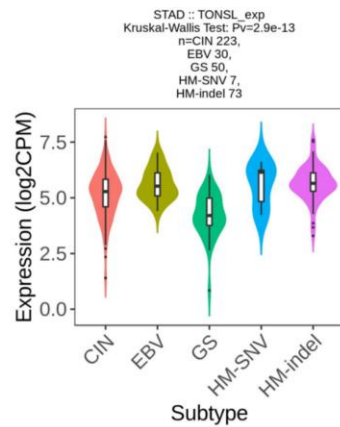

H

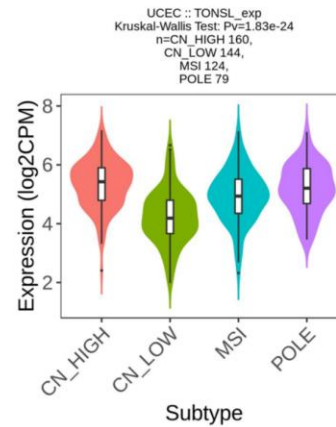

Supplement: Supplementary file 4 — Figure S4: Correlations between TONSL expression and molecular subtypes in 8 cancers where OS was not statistically significant. (A) BRCA, (B) COAD, (C) ESCA, (D) GBM, (E) HNSC, (F) LUSC, (G) STAD, (H) UCEC. [file CNR2-9-e70551-s001.pdf]

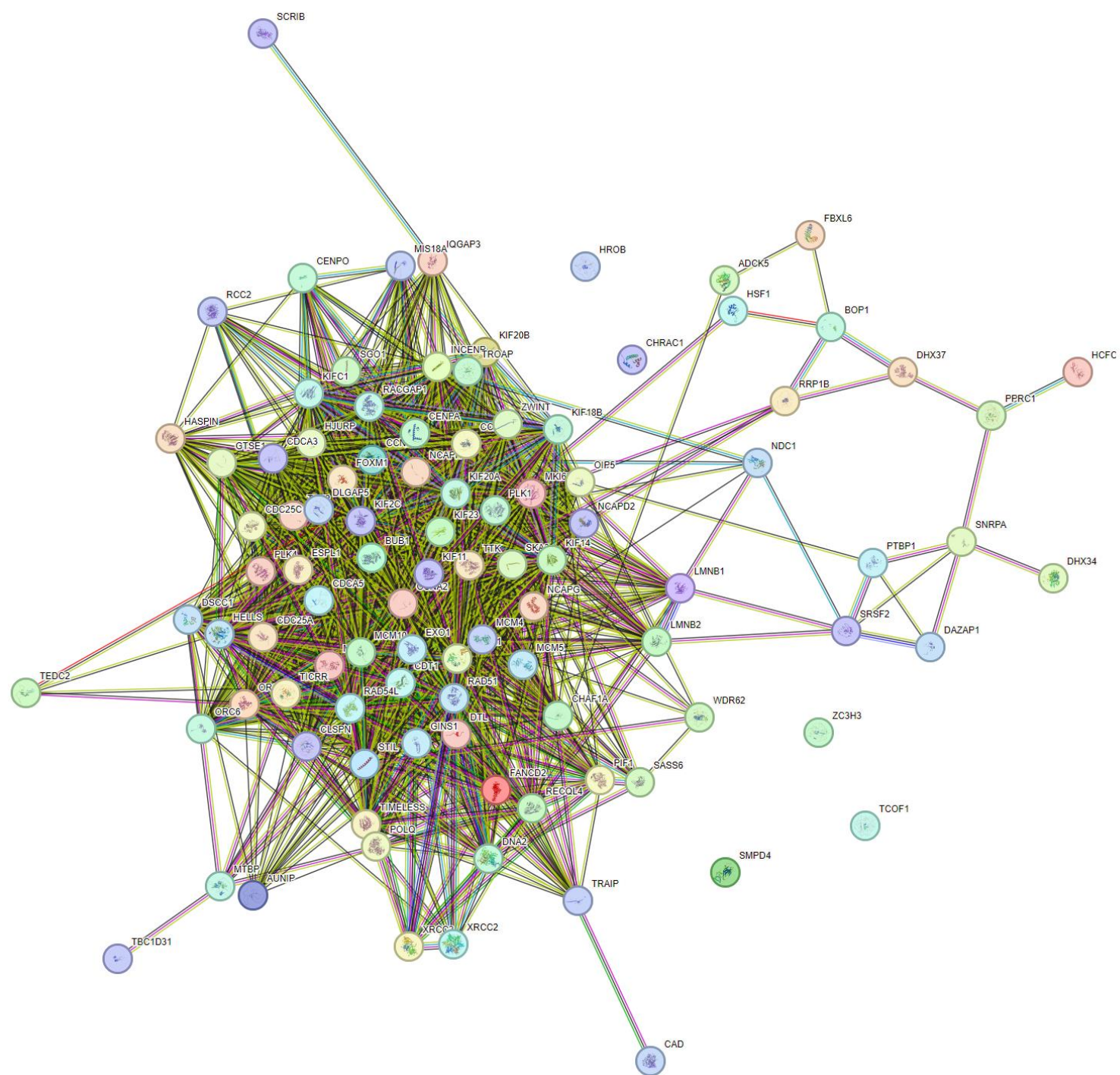

Supplement: Supplementary file 5 — Figure S5: The PPI network diagram based on 100 TONSL‐related genes from STRING database. [file CNR2-9-e70551-s005.pdf]
