# Supplementary material for: Comprehensive Bioinformatic Analysis of TONSL Expression in Pan‐Cancer
Source: Cancer Rep (Hoboken). 2026 Apr 19;9(4):e70551. doi: 10.1002/cnr2.70551 (PMC13092426; doi:10.1002/cnr2.70551)
Supplement: Supplementary file 6 — Table S1: Details of diagnostic ROC for TONSL in pan‐cancer. [file CNR2-9-e70551-s003.pdf]

Supplemental Table 1. Details of diagnostic ROC for TONSL in pan-cancer.

Tumor type	Tumor(n)	Normal(n)	AUC(CI)	cut-off	sensitivity	specificity	Positive predictive value	Negative predictive value	YI
**BLCA**	**412**	**19**	**0.935(0.860 - 1.000)**	**1.9504**	**0.87621**	**0.94737**	**0.99724**	**0.26087**	**0.82358**
**BRCA**	**1113**	**113**	**0.924(0.904 - 0.945)**	**1.3442**	**0.81042**	**0.9115**	**0.98904**	**0.32803**	**0.72193**
**COAD**	**480**	**41**	**0.923(0.899 - 0.948)**	**2.0807**	**0.84792**	**0.95122**	**0.99511**	**0.34821**	**0.79914**
**ESCA**	**163**	**11**	**0.922(0.856 - 0.988)**	**2.4008**	**0.80368**	**0.90909**	**0.99242**	**0.2381**	**0.71277**
GBM	169	5	0.882(0.825 - 0.938)	1.0813	0.82249	1	1	0.14286	0.82249
**HNSC**	**504**	**44**	**0.948(0.927 - 0.968)**	**1.7879**	**0.8254**	**0.97727**	**0.9976**	**0.32824**	**0.80267**
KICH	65	25	0.604(0.482 - 0.727)	0.62639	0.32308	0.92	0.91304	0.34328	0.24308
KIRC	541	72	0.744(0.692 - 0.797)	0.81627	0.59519	0.79167	0.95549	0.20652	0.38686
KIRP	291	32	0.820(0.757 - 0.883)	1.2278	0.65636	0.90625	0.98454	0.22481	0.56261
**LIHC**	**374**	**50**	**0.983(0.973 - 0.993)**	**0.71444**	**0.92513**	1	**1**	**0.64103**	**0.92513**
**LUAD**	**539**	**59**	**0.967(0.953 - 0.981)**	**1.1259**	**0.8961**	**0.9661**	**0.99588**	**0.50442**	**0.86221**
**LUSC**	**502**	**49**	**0.987(0.979 - 0.996)**	**1.294**	**0.96215**	**1**	1	**0.72059**	**0.96215**
PAAD	179	4	0.774(0.591 - 0.957)	1.0592	0.82123	0.75	0.99324	0.085714	0.57123
**PCPG**	**184**	**3**	**0.922(0.837 - 1.000)**	**0.96506**	**0.84783**	**1**	**1**	**0.096774**	**0.84783**
PRAD	501	52	0.870(0.823 - 0.917)	1.2099	0.79242	0.86538	0.98267	0.30201	0.6578
**READ**	**167**	**10**	**0.958(0.911 - 1.000)**	**1.7951**	**0.92814**	**0.9**	**0.99359**	**0.42857**	**0.82814**

**SARC**	**263**	**2**	**0.962(0.893 - 1.000)**	**0.93572**	**0.92776**	**1**	**1**	**0.095238**	**0.92776**
**STAD**	**375**	**32**	**0.925(0.896 - 0.955)**	**2.2372**	**0.75467**	**1**	**1**	**0.25806**	**0.75467**
THCA	512	59	0.809(0.763 - 0.855)	1.3824	0.71094	0.83051	0.97326	0.24873	0.54145
THYM	120	2	0.546(0.012-1.000)	1.7367	0.81667	0.5	0.9899	0.043478	0.31667
**UCEC**	**554**	**35**	**0.947(0.919 - 0.975)**	**1.8092**	**0.85199**	**0.91429**	**0.99368**	**0.2807**	**0.76627**

Abbreviations:ROC: Receiver Operator Characteristic curve; AUC: Area Under Curve; CI: Confidence Interval; YI: Youden’s indx.
